# Bacterial size matters: Multiple mechanisms controlling septum cleavage and diplococcus formation are critical for the virulence of the opportunistic pathogen *Enterococcus faecalis*

**DOI:** 10.1371/journal.ppat.1006526

**Published:** 2017-07-24

**Authors:** Bartłomiej Salamaga, Tomasz K. Prajsnar, Ana Jareño-Martinez, Joost Willemse, Martin A. Bewley, Françoise Chau, Tassadit Ben Belkacem, Annemarie H. Meijer, David H. Dockrell, Stephen A. Renshaw, Stéphane Mesnage

**Affiliations:** 1 Krebs Institute, University of Sheffield, Sheffield, United Kingdom; 2 Department of Molecular Biology and Biotechnology, University of Sheffield, Sheffield, United Kingdom; 3 Department of Infection and Immunity and Cardiovascular Disease, University of Sheffield, Sheffield, United Kingdom; 4 The Bateson Centre, University of Sheffield, Sheffield, United Kingdom; 5 Institute of Biology, Leiden University, Leiden, The Netherlands; 6 INSERM, UMR 1137, Infection, Antimicrobiens Modelisation, Evolution (IAME), Université Paris Diderot, Sorbonne Paris cité, Paris, France; Stanford University, UNITED STATES

## Abstract

*Enterococcus faecalis* is an opportunistic pathogen frequently isolated in clinical settings. This organism is intrinsically resistant to several clinically relevant antibiotics and can transfer resistance to other pathogens. Although *E*. *faecalis* has emerged as a major nosocomial pathogen, the mechanisms underlying the virulence of this organism remain elusive. We studied the regulation of daughter cell separation during growth and explored the impact of this process on pathogenesis. We demonstrate that the activity of the AtlA peptidoglycan hydrolase, an enzyme dedicated to septum cleavage, is controlled by several mechanisms, including glycosylation and recognition of the peptidoglycan substrate. We show that the long cell chains of *E*. *faecalis* mutants are more susceptible to phagocytosis and are no longer able to cause lethality in the zebrafish model of infection. Altogether, this work indicates that control of cell separation during division underpins the pathogenesis of *E*. *faecalis* infections and represents a novel enterococcal virulence factor. We propose that inhibition of septum cleavage during division represents an attractive therapeutic strategy to control infections.

## Author summary

*Enterococcus faecalis* is a commensal bacterium that colonizes the gastrointestinal tract of humans. This organism is an opportunistic pathogen that can cause a wide range of life-threatening infections in hospital settings. Despite the identification of several virulence factors, the mechanisms by which *E*. *faecalis* evades host immunity and causes infections remains poorly understood. Here, we explore how the formation of diplococci and short cell chains, a distinctive property of *E*. *faecalis*, contributes to pathogenesis. We describe several mechanisms that control the activity of AtlA, the enzyme dedicated to septum cleavage during division. Using a combination of *in vitro* assays and flow cytometry analyses of *E*. *faecalis* mutants, we show that AtlA activity is regulated by several mechanisms. We reveal that during pathogenesis, AtlA activity is critical for overcoming the host immune response. In the absence of AtlA, the long cell chains of *E*. *faecalis* mutants are more susceptible to phagocytosis and can no longer cause lethality in the zebrafish model of infection, thus indicating that control of cell chain length is a novel virulence factor in *E*. *faecalis*. This work highlights a link between cell division and pathogenesis and suggests that cell separation represents a step that can be targeted to control bacterial infections.

## Introduction

Enterococci are Gram-positive commensal bacteria colonizing the gastrointestinal tract of humans. They are opportunistic nosocomial pathogens that can cause a wide range of life-threatening infections in immunocompromised patients or following antibiotic-induced dysbiosis [1]. The emergence of enterococci as nosocomial pathogens relies on the capacity of these bacteria to colonize the host and to grow in a wide range of harsh conditions (*e*.*g*., in the presence of bile salts or in iron-depleted environments) [2]. Enterococci are intrinsically resistant to multiple antibiotics, such as cephalosporins and several aminoglycosides, and can also acquire resistance to glycopeptides. Vancomycin-Resistant Enterococci (VRE) represent a major problem in clinical settings as they can potentially transfer resistance genes to other pathogens such as *Staphylococcus aureus* [3, 4].

Two enterococcus species, *Enterococcus faecium* and *Enterococcus faecalis* are the most clinically relevant [5]. *E*. *faecium* infections are caused by a particular subset of clones specifically found in hospital settings that share several acquired mobile genetic elements [6]. In contrast, the *E*. *faecalis* strains responsible for hospital-acquired infections are also found in healthy individuals and genes associated with virulence are not only exclusively present in clinical isolates [7]. How this organism can cause infections therefore remains poorly understood.

In the present work, we study the regulation of daughter cell separation during cell division and explore the impact of this process on pathogenesis. We previously revealed that in *E*. *faecalis*, one peptidoglycan (PG) hydrolase with *N*-acetylglucosaminidase activity (named AtlA) is dedicated to septum cleavage to allow separation of daughter cells at the end of division [8]. Using a combination of *in vitro* experiments and sets of isogenic strains, we describe multiple mechanisms controlling the activity of AtlA. We show that control of septum cleavage during growth underpins the formation of diplococci and short chains, a property critical to cause lethality in the zebrafish model of infection.

## Results

### High expression levels of septum hydrolytic enzymes are not sufficient for cell separation

**Fig 1:**
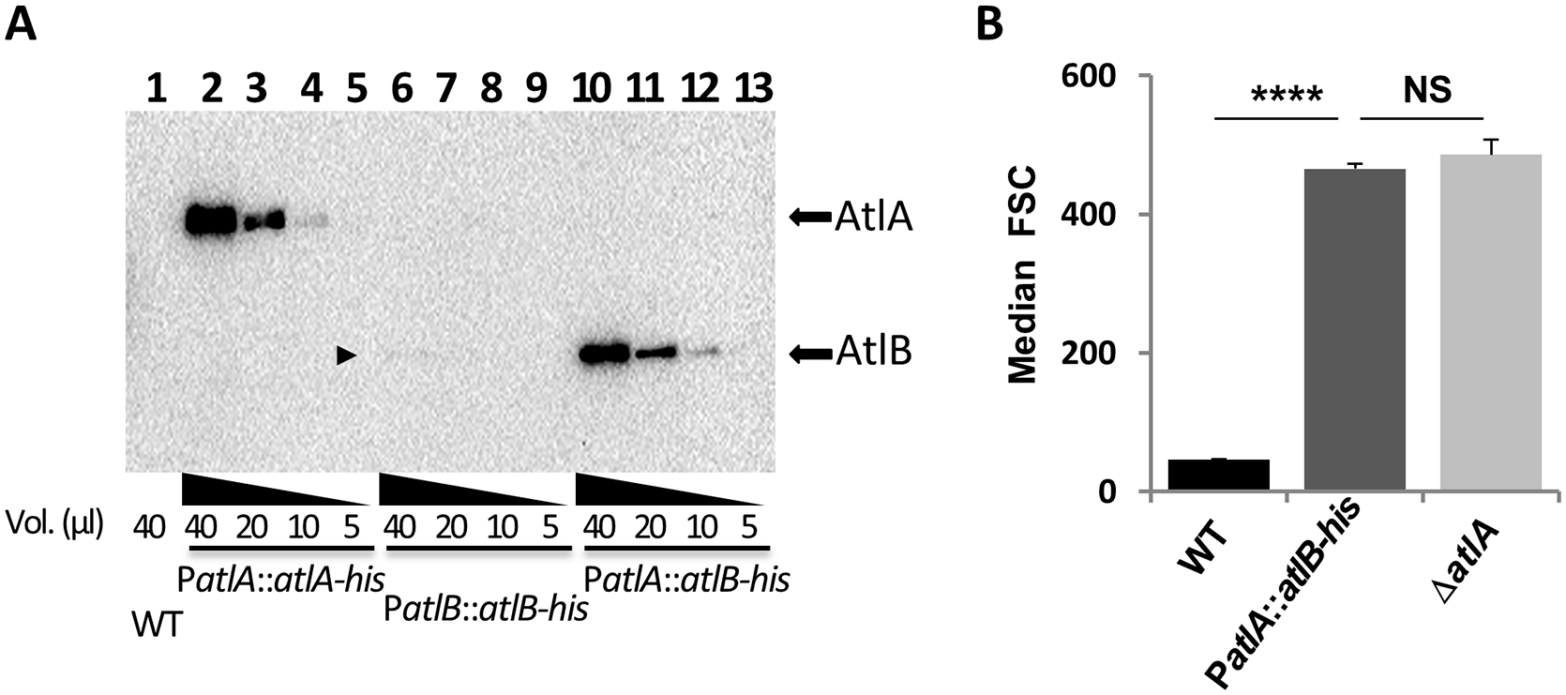
*E*. *faecalis* expressing *atlB* under the P*atlA* promoter forms cell chains. **A**. Western blot detection of His-tagged AtlA and AtlB proteins expressed under the control of the *atlA* (P*atlA*) or *atlB* (P*atlB*) promoters. Various amounts of exponentially growing cultures were harvested and total extracts corresponding to mixtures of broken cells and supernatants were analyzed by Western blot using anti-His antibodies. The faint band corresponding to AtlB-His is indicated by an arrowhead. **B**. Comparison of median forward scattered (FSC) light values corresponding to the cell chain lengths of WT, Δ*atlA* and P*atlB*::*atlB-his* strains; ****P<0.0001; n = 3; NS, *P*>0.05; n = 3.

*In vitro* enzymatic assays using recombinant *E*. *faecalis* PG hydrolases indicated that AtlA specific activity is 20- to 30-fold lower than the activity of the *N*-acetylmuramidase AtlB [9]. We hypothesized that a high level of AtlA expression could explain the predominant role of this enzyme in septum cleavage. To test this hypothesis, we compared the amount of AtlA and AtlB produced during growth. We generated two strains producing His-tagged AtlA and AtlB proteins expressed under their own promoters (P*atlA*::*atlA*-*his* and P*atlB*::*atlB*-*his*). Culture samples were harvested at the end of exponential growth (OD_600_ = 1) and His-tagged AtlA and AtlB were detected by western blotting using an anti-histidine serum (Fig 1A). Unlike His-tagged AtlA, AtlB was barely detectable (Fig 1A, lanes 2–5 and lanes 6–9; see arrowhead on Fig 1A). Next, we tested whether increasing the level of expression of AtlB could enable this peptidoglycan hydrolase to cleave the septum efficiently. We built a strain producing an AtlB-His tagged protein expressed under the *atlA* promoter (P*atlA*::*atlB*-*his*), thereby replacing the AtlA open reading frame by AtlB. As expected, expression of *atlB-his* under the control of the *atlA* promoter increased the production of AtlB-His to levels similar to AtlA-His (Fig 1A, compare lanes 2–5 with lanes 10–13). The impact of AtlB production on septum cleavage was analyzed by flow cytometry to measure bacterial chain lengths, as previously described [8]. Increasing the production of AtlB to levels similar to those of AtlA was not sufficient to shorten bacterial cell chains (Fig 1B). The cell chain length of the P*atlA*::*atlB-his* strain was not significantly different from that of the Δ*atlA* strains (*P*>0.05; n = 3). This result indicated that the relatively low production level of AtlB does not account for the low septum cleavage activity of this enzyme. This prompted us to explore the enzymatic properties of AtlA and their impact on cell separation.

### N-terminal proteolytic cleavage of recombinant AtlA stimulates septum cleavage

**Fig 2:**
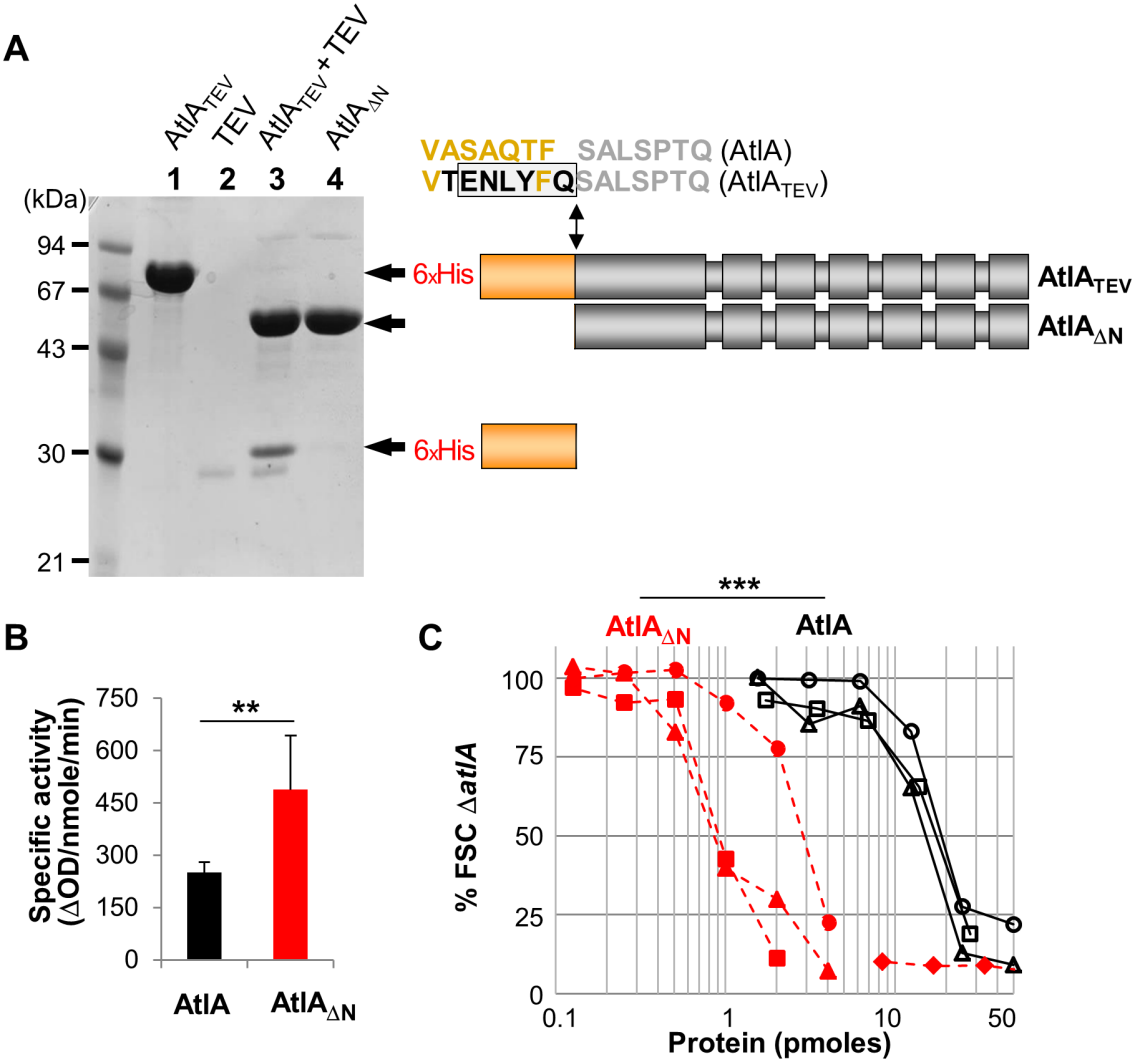
Truncation of AtlA N-terminal domain enhances septum cleavage activity *in vitro*. **A**. SDS-PAGE analysis of purified recombinant proteins: lane 1, full-length AtlA_TEV_ (residues 53–737) corresponding to the mature protein (without the signal peptide), with a TEV site between domains 1 and 2; lane 2, TEV protease (TEV); lane 3, TEV digestion product of AtlA_TEV_ (AtlA_TEV_+TEV); lane 4, AtlA truncated of its N-terminal domain (AtlA_ΔN_). The amino acid sequences between N-terminal (orange) and catalytic (grey) domains in AtlA and AtlA_TEV_ are described. The TEV cleavage site is boxed. **B**. Comparison of specific enzymatic activities of AtlA and AtlA_ΔN_ *in vitro* using whole PG sacculi as a substrate; **P = 0.0018; n = 9. **C**. Flow cytometry analysis of septum cleavage activity of recombinant full-length AtlA and the N-terminally truncated variant (AtlA_ΔN_). Activity is expressed as a percentage of the median forward scattered (FSC) light value corresponding to cell chains formed by the *atlA* mutant (Δ*atlA*) used as a substrate; ***P = 0.0008; n = 3.

Previous studies indicated that truncation of the AtlA N-terminal domain (residues 54 to 172) only had a marginal impact on the activity of the recombinant enzyme tested *in vitro* against whole PG molecules (sacculi) as a substrate [10]. We sought to re-investigate the contribution of the N-terminal domain to AtlA activity using an *in vitro* assay (as described in [8]) to specifically measure septum cleavage. A recombinant AtlA protein (residues 54 to 737) harboring an N-terminal 6-Histidine tag and a Tobacco Etch Virus (TEV) protease site at the end of the N-terminal domain was expressed in *Escherichia coli* and purified (Fig 2A, lane 1). Following cleavage with TEV protease, truncated AtlA was recovered by metal affinity chromatography (Fig 2A, lanes 2 to 4). In agreement with previous results, the specific activity of the truncated AtlA (AtlA_ΔN_; 488.2 ± 155.6 ΔOD_600_/nmole/min) was slightly higher than that of AtlA (252.5 ± 27.4 ΔOD_600_/nmole/min) when assayed against whole sacculi (**P = 0.0018; n = 9; Fig 2B). However, using the specific septum cleavage assay, AtlA_ΔN_ was more than 10-fold active than the full-length AtlA enzyme. Whilst 1.6 ± 1.2 pmoles of AtlA_ΔN_ were sufficient to disperse 50% of the cell chains, 18.2 ± 2.0 pmoles of AtlA were required for a similar septum cleavage activity (***P = 0.0008; n = 3; Fig 2C).

### Cleavage of AtlA N-terminal domain is required for daughter cell separation during growth

**Fig 3:**
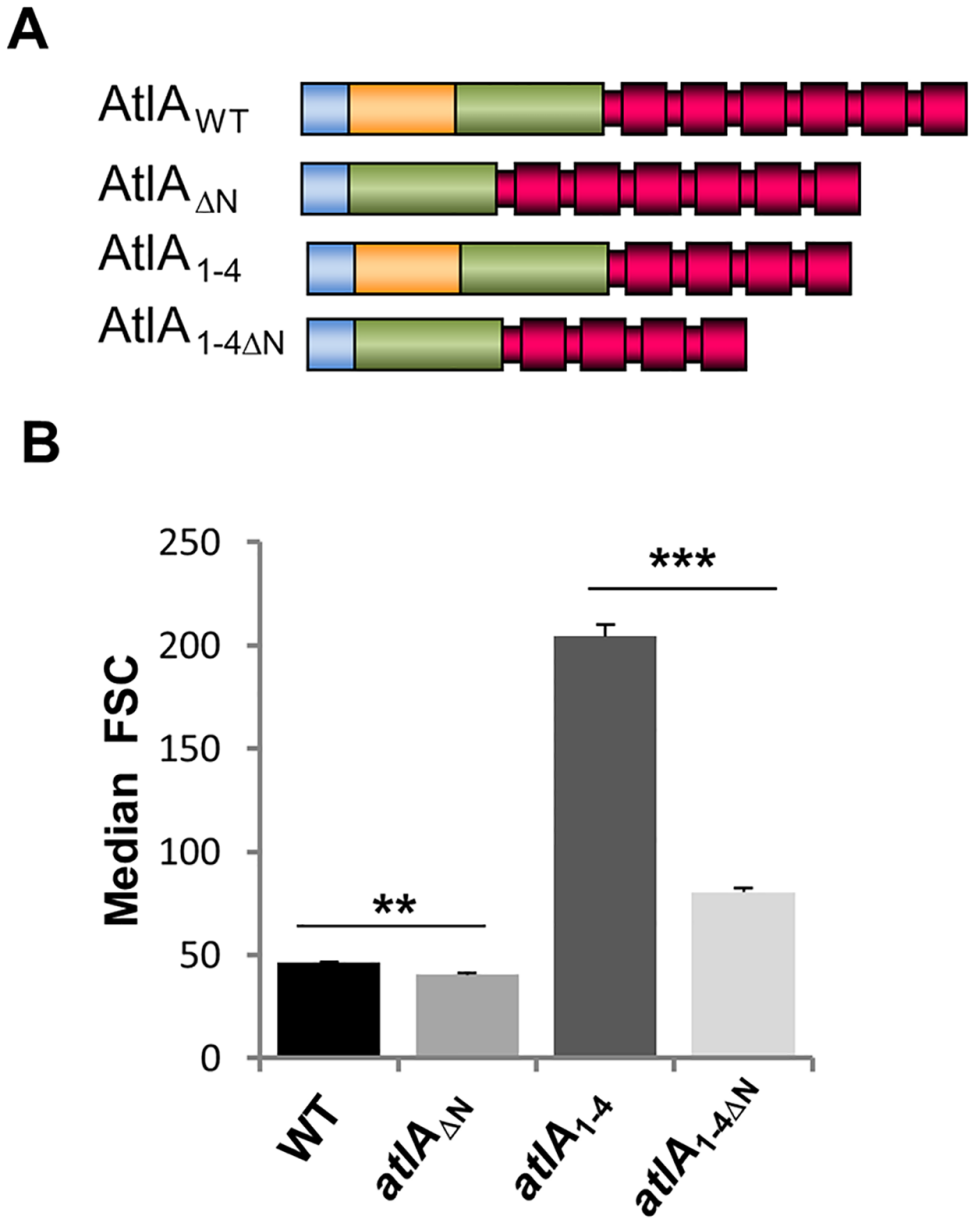
*E*. *faecalis* strains expressing N-terminally truncated AtlA form shorter cell chains. **A**. Schematic representation of AtlA variants produced by recombinant *E*. *faecalis* JH2-2 derivatives. AtlA_WT_, full-length AtlA; AtlA_ΔN_, AtlA truncated from the N-terminal domain; AtlA_1-4_, AtlA truncated from the two C-terminal LysM modules; AtlA_1-4ΔN_, AtlA_1-4_ truncated from the N-terminal domain. Blue, signal peptide; orange, N-terminal domain; green, catalytic domain; red, LysM domain. **B**. Comparison of median forward scattered (FSC) light values corresponding to the cell chain lengths of WT, *atlA*_ΔN_, *atlA*_1-4_ and atlA_1-4ΔN_ strains; **P = 0.0015; n = 3; ***P = 0.0001; n = 3. See also S2 Fig.

We further explored the contribution of the AtlA N-terminal domain in septum cleavage during growth. We built a recombinant strain producing AtlA with a truncated N-terminal domain (*atlA*_ΔN_) (Fig 3A). However, as the parental strain mostly forms diplococci and short chains (2–4 cells), we anticipated only a limited reduction in bacterial cell chain length. To see a more pronounced reduction in bacterial chain length, we analyzed the impact of the N-terminal truncation in a strain forming longer chains. We suspected that truncating the C-terminal domain of AtlA would impair binding of this enzyme to its substrate and its activity, hence leading to the formation of longer chains. We therefore constructed *atlA*_1-4_, a strain producing an AtlA variant with only 4 C-terminal LysM repeats instead of 6. AtlA proteins produced by recombinant strains were detected in culture supernatants by Western blot using polyclonal antibodies raised against the catalytic domain of AtlA, indicating that AtlA proteins with the expected molecular weights were produced and secreted in all cases (S1A Fig). The distribution of cell chain lengths measured by flow cytometry was in agreement with our *in vitro* experiments. Truncation of the N-terminal domain led to the formation of shorter chains as indicated by a significant shift towards lower forward scattered light values (Fig 3B). This conclusion was supported by two pairwise comparisons (i) between the cells producing full-length AtlA (WT; FSC = 46.38 ± 0.38) and its N-terminally truncated counterpart (*atlA*_ΔN_; FSC = 40.32 ± 0.99) (**P = 0.0015; n = 3) and (ii) between the cells producing AtlA with 4 C-terminal LysM repeats (*atlA*_1-4_; FSC = 204.45 ± 5.71) and its N-terminally truncated counterpart (*atlA*_1-4ΔN_; FSC = 80.33 ± 1.99) (***P = 0.0001; n = 3). The reduction of cell numbers per chain in the mutants producing a truncated AtlA protein was confirmed by light microscopy (S1B and S1C Fig) Altogether, these results showed that the N-terminal domain of AtlA negatively controls the septum cleavage activity of this enzyme.

### AtlA N-terminal domain glycosylation inhibits septum cleavage

**Fig 4:**
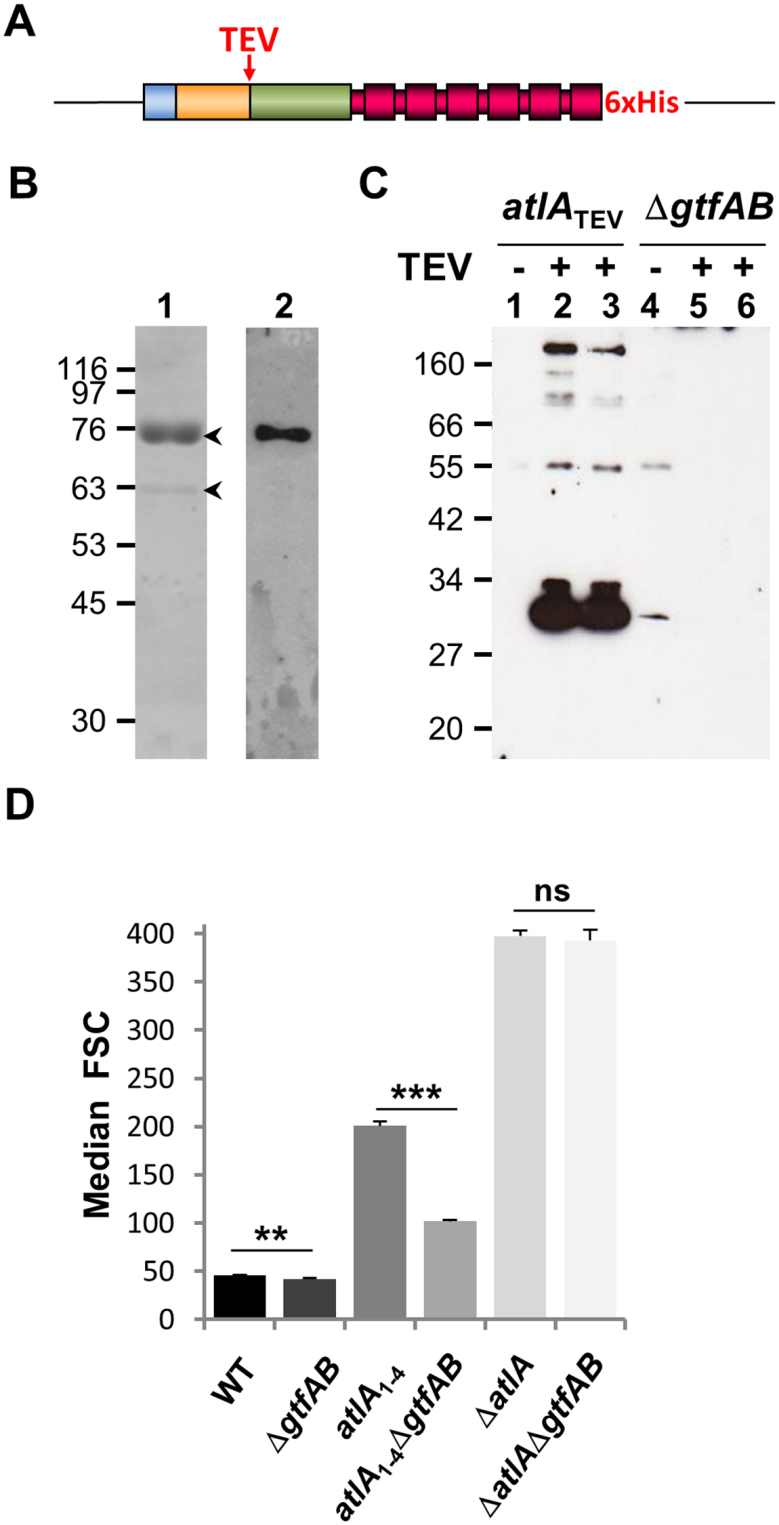
*E*. *faecalis* strains lacking AtlA glycosylation form shorter cell chains. **A**. Schematic representation of the *atlA*_TEV_ allele produced by *E*. *faecalis*. **B**. Metal affinity purification of cell surface associated His-tagged AtlA extracted with 8M urea. Two bands indicated by arrowheads were detected on a Coomassie-stained SDS-PAGE (lane 1); the upper band (72 kDa) corresponds to full-length AtlA proteins and the lower band to AtlA without the N-terminal domain (62 kDa). A clear signal corresponding to glycosylated full length AtlA (lane 2) was detected using the ECL glycoprotein detection kit (GE Healthcare). **C**. Exponentially growing cells from a culture expressing AtlA_TEV_ were resuspended in buffer in the absence (-) or presence (+) of TEV protease to cleave the N-terminal domain of AtlA. Solubilized proteins were recovered by centrifugation, loaded on an SDS-PAGE and transferred on nitrocellulose to detect glycosylated proteins. Two independent cultures treated with the TEV protease were analysed in parallel. In both cases, a glycosylated polypeptide with the expected molecular weight for the N-terminal domain (see Fig 2A) was detected while no signal was observed in the negative control. When a similar experiment was repeated with protein extracts from a Δ*gtfAB* mutant, no glycosylated protein was detected, indicating that this operon is involved in the post translational modification of AtlA. **D**. Comparison of median forward scattered (FSC) light values corresponding to the cell chain lengths of WT, Δ*gtfAB*, *atlA*_1-4_, *atlA*_1-4_Δ*gtfAB*, Δ*atlA* and Δ*atlA*Δ*gtfAB* strains.

The N-terminal domain of AtlA contains a high proportion of threonine and serine residues (28% and 12%, respectively; S1D Fig). This property prompted us to test whether this domain can be *O*-glycosylated. To purify AtlA produced by *E*. *faecalis*, a recombinant strain expressing a C-terminally 6His-tagged AtlA protein and a TEV site between the N-terminal and catalytic domains was constructed by allele exchange (Fig 4A). Cell surface proteins were extracted with 8M urea and His-tagged AtlA protein was purified by metal affinity chromatography. Two major bands of 75 kDa and 62 kDa matching the expected molecular weights of the full-length and N-terminally truncated AtlA, respectively, were detected (Fig 4B, lane 1). A carbohydrate moiety was detected on the full-length AtlA protein, but absent on the truncated AtlA (Fig 4B, lane 2), suggesting that glycosylation occurred at the N-terminal domain of AtlA. To confirm this hypothesis, exponentially growing cells were harvested and incubated in the presence of TEV protease. This treatment released a glycosylated polypeptide matching the apparent molecular weight of the N-terminal domain (Fig 4C, lanes 2 and 3 and Fig 2A, lane 3). No glycosylated polypeptide was detected when the protease was omitted, therefore indicating that the N-terminal domain of AtlA is glycosylated. In Gram-positive bacteria, two glycosyl transferases named GtfA and GtfB have been shown to be essential for surface protein glycosylation [11–13]. We used allele exchange to inactivate two putative glycosyl transferase homologs (*gtfA* and *gtfB*; EF2891 and EF2892 in *E*. *faecalis* V583) sharing the same glycosyl transferase domain (PFAM PF00534). Following incubation of cells harboring an in-frame deletion of the *gtfAB* locus in the presence of TEV protease, no glycosylated peptide could be detected (Fig 4C, lanes 4–6). Altogether, these results show that the N-terminal domain of AtlA is glycosylated and that this posttranslational modification requires the glycosyltransferases *gtfAB*. Next, we explored the impact of AtlA glycosylation on septum cleavage during growth by measuring the bacterial chain length of *gtfAB* mutants by flow cytometry (Fig 4D) and light microscopy (S2 Fig). We compared the cell chain lengths of *E*. *faecalis* JH2-2 (WT) forming mostly diplococci and short chains (2–4 cells) and *atlA*_1-4_, producing AtlA_1-4_ lacking two LysM modules (6–12 cells) with the chain length of their Δ*gtfAB* counterparts. Pairwise comparisons of cell chain length by flow cytometry revealed that Δ*gtfAB* mutants formed shorter chains than parental strains, thus indicating that the lack of glycosylation enhanced AtlA septum cleavage activity. The cell chains of the parental strain (WT; FSC = 46.09 ± 0.43) were longer than those from the Δ*gtfAB* mutant (FSC = 42.04 ± 0.66; **P = 0.0017; n = 3). A more pronounced difference was measured between the strain expressing the glycosylated C-terminally truncated AtlA (*atlA*_1-4_; FSC = 200.87 ± 5.71) and its non-glycosylated counterpart (*atlA*_1-4_Δ*gtfAB*; FSC = 101.66 ± 1.47) (***P = 0.0002; n = 3). When introduced to the Δ*atlA* genetic background, the deletion of the *gtfAB* operon did not significantly reduce the bacterial chain length (FSC = 397.68 ± 5.37 versus 392.95 ± 11.37, respectively; *P*>0.05). Together with light microscopy analyses (S2C and S2D Fig), these results indicate that AtlA glycosylation mediated by *gtfAB* impairs septum cleavage.

### Swapping AtlA *N*-acetylglucosaminidase domain for other hydrolase domains cleaving distinct peptidoglycan bonds does not abolish septum cleavage in *E*. *faecalis*

**Fig 5:**
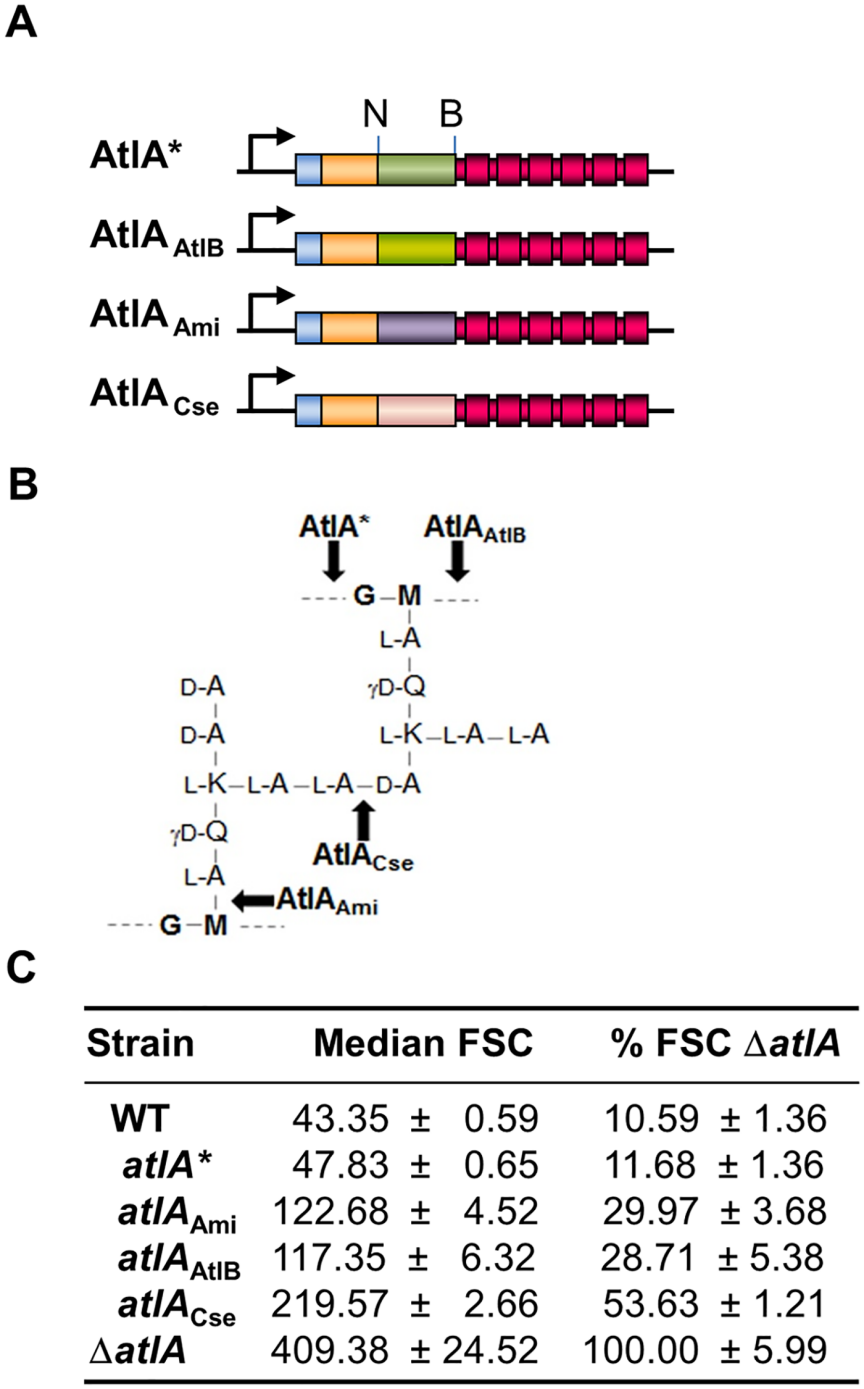
AtlA *N*-acetylglucosaminidase activity is not essential for septum cleavage. **A**. Schematic representation of AtlA variants expressed by recombinant *E*. *faecalis* JH2-2 derivatives. All strains were constructed by allele exchange to express AtlA variants under the control of the *atlA* promoter (arrow). Two restriction sites (NcoI, N and BglII, B) flanking the region encoding the catalytic domain were introduced by site-directed mutagenesis. The resulting allele in strain atlA* encodes an AtlA protein with eleven modified amino-acids (see supplementary S3 Fig). The NcoI-BglII fragment encoding AtlA *N*-acetylglucosaminidase activity was replaced with a fragment encoding the *N*-acetylmuramidase activity of *E*. *faecalis* AtlB to generate strain *atlA*_AtlB_, the amidase activity of *S*. *aureus* Atl to generate strain *atlA*_Ami_ or the endopeptidase activity of *Streptococcus thermophilus* Cse to generate strain *atlA*_Cse_. **B**. *E*. *faecalis* peptidoglycan bonds cleaved by the catalytic domains of *E*. *faecalis* AtlA and AtlB, *S*. *aureus* AtlA and *S*. *thermophilus* Cse. **C**. Comparison of median forward scattered (FSC) light values corresponding to the cell chain lengths of WT, atlA*, *atlA*_AtlB_, *atlA*_Ami_, *atlA*_Cse_. and Δ*atlA*. All median FSC values were significantly different from the median FSC value from the Δ*atlA* strain (**P<0.01; n = 3).

Substrate recognition by the catalytic domain of peptidoglycan hydrolases is an important factor modulating enzymatic activity [14]. The fact that AtlA is dedicated to septum cleavage can therefore be underpinned by the recognition and cleavage of a specific peptidoglycan structure present at the septum. We hypothesized that if such is the case, the *N*-acetylglucosaminidase activity of AtlA should be essential for septum cleavage. To explore this possibility, we constructed a gene replacement vector encoding an allele of *atlA* with a catalytic domain flanked by two restriction sites (NcoI and BglII). These two sites were used to swap the *N*-acetylglucosaminidase domain of AtlA for the catalytic domains of *E*. *faecalis N*-acetylmuramidase AtlB [8], *Staphylococcus aureus N*-acetylmuramoyl-L-Alanine amidase Atl [15] or *Streptococcus thermophilus* D,L endopeptidase Cse [16] (Fig 5A and S3 Fig). AtlA alleles encoding variants with distinct peptidoglycan cleavage specificities (Fig 5B) were introduced on the chromosome by gene replacement and expressed as a single copy under the *atlA* promoter. Western blot analyses indicated that all AtlA variants were expressed at similar levels, except AtlA_Cse_, which was subject to proteolysis (S3C Fig) The septum cleavage activity in each strain was analyzed using flow cytometry. Cell chain length of the Δ*atlA* deletion mutant was used as a reference to define the forward scattered light value corresponding to maximal (100%) chain length. Introduction of the NcoI and BamHI restriction sites had a limited impact on the size of the cell chains (11.68 ± 1.35% of the Δ*atlA* mutant chains versus 10.59 ± 1.36% of the Δ*atlA* mutant for the parental JH2-2 strain). All strains expressing AtlA variants with altered enzymatic specificity formed shorter chains than the Δ*atlA* mutant in exponential phase (Fig 5C). This result indicated that the peptidoglycan cleavage specificity of AtlA is not an essential property of the enzyme for septum cleavage. The forward scattered light measurements corresponding to the strains expressing AtlA variants compared to the Δ*atlA* mutant ranged from 28.71 ± 5.38% of the Δ*atlA* mutant for the strain expressing AtlB with a muramidase activity to 53.63 ± 1.3% of the Δ*atlA* mutant for the strain expressing AtlA with an endopeptidase activity. The relatively higher forward scattered light values associated with this strain could be due to the proteolysis of the chimeric protein (S3C Fig). The chain lengths of strains expressing chimeric proteins were all significantly shorter than those of the Δ*atlA* mutant (**P<0.01; n = 3).

### The multimodular LysM domain of AtlA is a major determinant for septum cleavage

**Fig 6:**
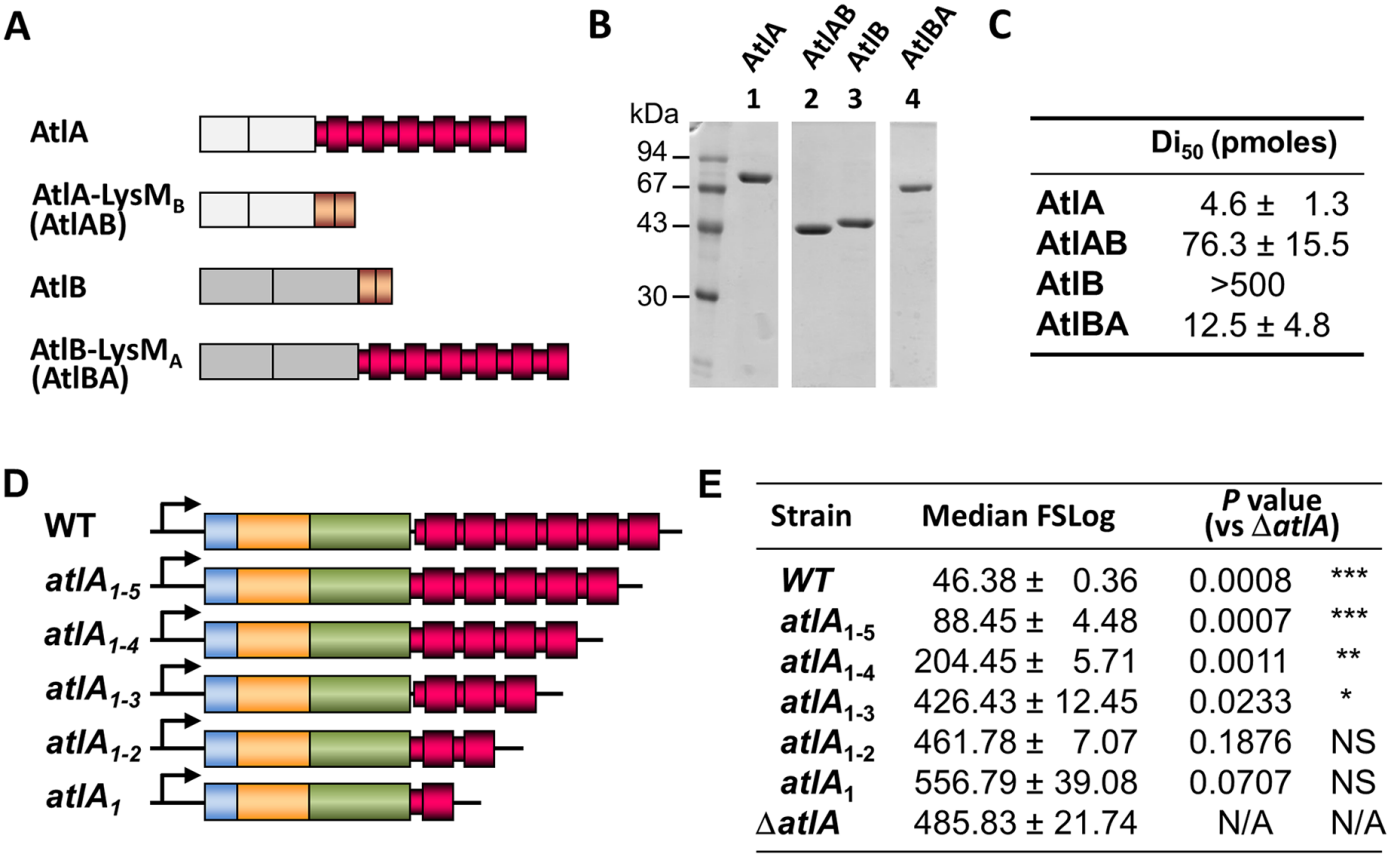
Contribution of the LysM domain to septum cleavage. **A**. Schematic representation of AtlA and AtlB derivatives expressed and purified to test their septum cleavage activity. Full-length AtlA and AtlB (without signal peptides), as well as their counterparts with LysM_B_ (AtlAB) or LysM_A_ (AtlBA) domains, were expressed in *E*. *coli*. **B**. SDS-PAGE of AtlA (lane 1), AtlAB (lane 2), AtlB (lane 3) and AtlBA (lane 4) samples showing that all proteins were purified to homogeneity. **C**. Flow cytometry analysis of septum cleavage activity of recombinant proteins *in vitro* using OG1RF Δ*atlA* cell chains as a substrate (see materials and methods). The Di_50_ (Dechaining index) value corresponds to the amount of enzyme in pmoles that is able to decrease the median FSC value of Δ*atlA* cell chains by 50% in 15 minutes at 37°C. **D**. Schematic representation of *atla* locus in *E*. *faecalis* JH2-2 and isogenic derivatives producing AtlA with a C-terminal LysM domain containing a variable number of LysM repeats (6 in WT; 5 in *atlA*_1-5_; 4 in *atlA*_1-4_; 3 in *atlA*_1-3_; 2 in *atlA*_1-2_; 1 in *atlA*_1_). **E**. Comparison of median forward scattered (FSC) light values corresponding to the cell chain lengths of WT, *atlA*_1-5_, *atlA*_1-4_, *atlA*_1-3_, *atlA*_1-2_, *atlA*_1_ and Δ*atlA* strains. *P* values and significance corresponding to comparisons with the Δ*atlA* strain are indicated.

To test the contribution of the LysM domain of AtlA (LysM_A_) to septum cleavage, we compared the septum cleavage activity of proteins containing this domain or the LysM domain from AtlB (LysM_B_) using flow cytometry. Four recombinant proteins were produced in *E*. *coli* and purified: the full-length AtlA and AtlB proteins (without a signal peptide) and derivatives containing a swapped LysM domain (AtlAB and AtlBA; Fig 6A and 6B; S4 Fig). In agreement with our previous work, AtlA septum cleavage activity was much higher than that of AtlB. Whilst 4.6 ± 1.3 pmoles of AtlA reduced the cell chain length of a Δ*atlA* mutant by 50% in 15 minutes at 37°C, 100-fold more AtlB was not sufficient to produce the same effect. When AtlA LysM_A_ was replaced by LysM_B_, the septum activity of the enzyme decreased 17-fold indicating that LysM_A_ is critical for optimal septum cleavage. Swapping LysM_B_ for LysM_A_ in AtlB led to a septum cleavage activity comparable to that of AtlA (Fig 6C).

The contribution of individual LysM repeats to the septum cleavage activity was investigated. A set of strains expressing AtlA with 1 to 5 LysM repeats (henceforth referred to as *atlA*_1_ to *atlA*_1-5_ strains) was constructed by allele exchange (Fig 6D). Western blot and zymogram analyses (S4A and S4B Fig) showed that all strains produced AtlA proteins with the expected size and in similar amounts, except for the *atlA*_1_strain in which lower amounts were detected. AtlA activity decreased as LysM repeats were truncated. Next, we measured the impact of LysM truncations on the septum cleavage activity by measuring the cell chain length. Flow cytometry analyses revealed that sequential truncation of LysM modules led to a stepwise increase of cell chain length (Fig 6E and S4C Fig). This result indicated that optimal septum cleavage requires the full-length LysM domain, each module providing an additive contribution to AtA enzymatic activity.

### Cell separation defects abolish *E*. *faecalis* virulence in the zebrafish model of infection

**Fig 7:**
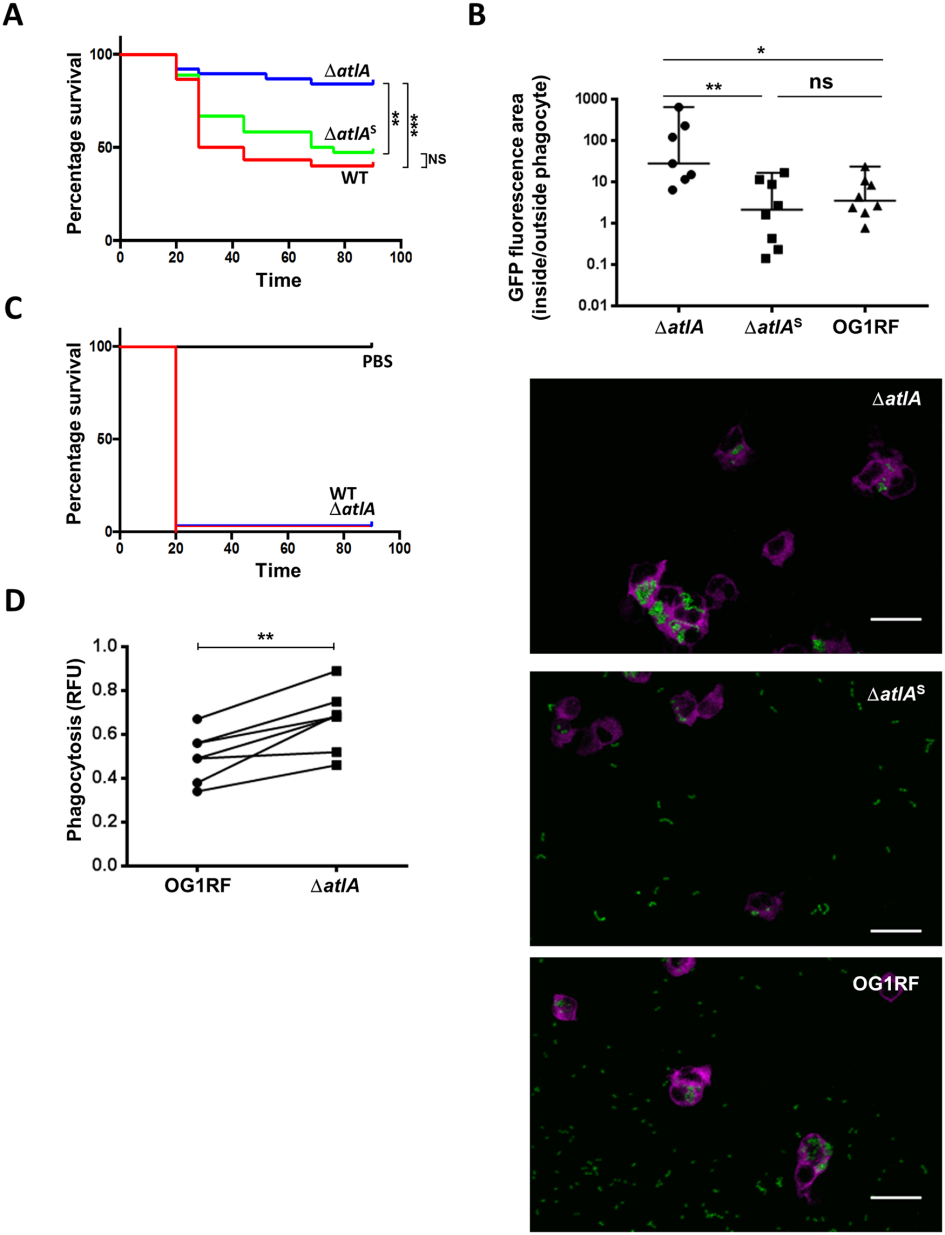
*E*. *faecalis* long cell chains are less virulent in the zebrafish model of infection and more prone to phagocytosis than diplococci. **A**. Survival of zebrafish larvae (n>20) following infection with *E*. *faecalis* OG1RF (WT) and *atlA* isogenic deletion mutant before (Δ*atlA*) and after (Δ*atlA*^S^) sonication to disperse long chains. Statistical significance was determined by Log-rank test; **P = 0.0011; *** *P* = 0.0002; NS, *P*>0.05. **B**. Quantification of *E*. *faecalis* uptake by zebrafish phagocytes. Embryos were infected with 1,200 *E*. *faecalis* cells expressing GFP and fixed in 4% paraformaldehyde 1.5h post infection. Phagocytes were immunolabelled using rabbit anti L-plastin antibodies and detected with goat anti-rabbit antibodies conjugated to Alexafluor 647. Fluorescent bacteria and phagocytes were imaged by scanning confocal microscopy. The area of GFP fluorescence signal outside and inside phagocytes was measured using a dedicated Fiji plugin. The ratio of GFP fluorescence area outside to inside phagocytes was used to quantify bacterial uptake. Phagocytosis was significantly higher for long chains (Δ*atlA*) when compared to their sonicated counterparts (Δ*atlA*^S^) (**P = 0.0098) or the wild-type cells (*P = 0.0438). No difference in uptake was found between short chains corresponding to the wild-type or sonicated Δ*atlA* mutant (NS, *P*>0.05). Representative images of phagocytes (magenta) following infection with Δ*atlA*, sonicated Δ*atlA*^S^ and wild-type OG1RF cells shown. Phagocytes labeled with L-plastin appear in magenta, GFP-producing bacteria in green. Scale bar is 20μm. **C**. Survival of phagocyte-depleted zebrafish larvae (n>20) following injection with *E*. *faecalis* OG1RF (WT) or Δ*atlA*. **D**. Pairwise comparisons of phagocytosis indexes corresponding to *E*. *faecalis* OG1RF and Δ*atlA* uptake by human monocyte-derived macrophages (MDM). Statistical significance was determined by paired t-test; Δ*atlA* cells were more efficiently phagocytosed by MDM than WT cells (**P = 0.0024; n = 7).

We investigated whether the formation of long chains in *E*. *faecalis* has an impact on virulence using the zebrafish model of infection [17]. We compared the lethality induced by the wild-type OG1RF strain to that of an in-frame *atlA* deletion mutant OG1RF (Δ*atlA*) forming long chains. In the Δ*atlA* mutant, each chain (equivalent to 1 CFU) can contain several viable cells. To eliminate this bias, Δ*atlA* chains were sonicated to mechanically separate cells (S5A and S5B Fig) [18] and establish the number of cells per CFU. This information was then used to inject the same number of cells (but different CFU numbers) for each strain. Zebrafish embryos were infected 30h post fertilization (hpf) and survival was monitored over the following 90h. One of three independent experiments is shown in Fig 7A whilst the results for three biological replicates are shown in S5C and S5D Fig. Injection of *ca*. 1,000 OG1RF cells killed between 60% and 77% of larvae (n = 79) depending on the experiment. In contrast, the Δ*atlA* mutant only killed 4% to 20% of larvae (n = 88), showing a significant reduction in virulence (***P<0.001 for the experiment shown in Fig 7A). At this stage, we envisaged that this difference could be attributed to several factors: (i) the increased chain length of the Δ*atlA* mutant, (ii) (an)other alteration(s) in cell surface properties (like in the *Streptococcus mutans atlA* mutant; [19]) or (iii) a defect in biofilm formation [20]. To specifically investigate the contribution of bacterial chain length to virulence, we subjected the Δ*atlA* mutant to mild sonication. This treatment is dispersing bacterial chains (S5A Fig) whilst it does not alter viability (Dubee et al., 2011), virulence (S6 Fig) or subsequent bacterial growth rate (S7 Fig). Sonication of the Δ*atlA* mutant thus allowed us to compare cells with an identical genetic background, differing only by the size of their cell chains. This treatment restored the virulence of the mutant to similar levels as the wild-type strain (*P* = 0.455; Fig 7A), killing between 46% and 59% of larvae (n = 90).

Previous work revealed that phagocyte evasion is a critical step for *E*. *faecalis* pathogenesis in the zebrafish [17]. We therefore quantified phagocytosis in zebrafish larvae infected with bacteria expressing the green fluorescent protein (GFP) (Fig 7B). Confocal microscopy images were used to measure bacterial uptake by phagocytes labelled with anti L-plastin antibodies. To specifically investigate the impact of the chain forming phenotype on phagocytosis, we compared the uptake of long and short bacterial chains formed by the Δ*atlA* mutant before and after sonication (Δ*atlA*^S^). The ratio between green fluorescence area inside to outside phagocytes was significantly higher for the Δ*atlA* mutant (**P = 0.0098; n = 7). A significant difference was also measured when we compared the uptake of the Δ*atlA* mutant to that of wild-type (*P = 0.0438; n = 8). As expected, no difference was detected between wild-type and Δ*atlA*^S^ cells. Representative images used to quantify uptake are shown as an example (Fig 7B). Bacteria forming long chains were mostly found inside phagocytes (96% of total fluorescence area) as opposed to diplococci and short chains corresponding to wild-type and Δ*atlA*^S^ cells (75% and 60% respectively).

Death rates were the same when immunocompromised zebrafish (n>20 per group) were infected with the wild-type OG1RF strain or with the Δ*atlA* mutant. In both cases, injection of 1250 cells in *pu*.*1* morphants led to more than 90% mortality within 20h post infection (Fig 7C and S5E Fig). This result suggested that the impaired virulence of the long chain-forming Δ*atlA* mutant is due to the fact that this strain is unable to evade phagocytosis during infection. To make a direct comparison of the uptake of wild-type and Δ*atlA* cells by phagocytes, an *in vitro* assay was performed using monocyte-derived macrophages obtained from peripheral blood mononuclear cells from healthy volunteers (Fig 7D). Phagocytic uptake was quantified following labeling of bacterial cells with pHrodo-S-ester, a pH-sensitive dye displaying increased fluorescence in low-pH compartments of phagosomes [21]. In each pairwise comparison, Δ*atlA* long chains were more efficiently phagocytosed (*P* = 0.0024 over 7 biological replicates) (Fig 7D). Collectively, our results indicate that formation of short chains by *E*. *faecalis* is a critical property enabling this bacterium to evade phagocytosis during pathogenesis.

## Discussion

The formation of diplococci and short chains is a distinctive property of *E*. *faecalis* that was originally reported over a century ago [22]. This typical morphology results from the separation of daughter cells by the *N*-acetylglucosaminidase AtlA [8]. Here, we show that multiple mechanisms are in place to control septum cleavage by AtlA. We further demonstrate that the formation of diplococci and short chains is crucial for the virulence of this opportunistic pathogen. Cells with impaired cell separation are more prone to phagocytosis and can no longer cause infection. We propose that the control of cell chain length is a novel virulence factor that links cell division and pathogenesis in *E*. *faecalis*.

Using a specific assay to measure septum cleavage by flow cytometry, we showed that two post-translational modifications, both occurring on the N-terminal domain of AtlA contribute to down-regulate the activity of this enzyme. Our hypothesis is that the N-terminal domain, predicted to be disordered (http://prdos.hgc.jp), sterically hinders the catalytic activity of AtlA. By extension, glycosylation of the N-terminal domain is expected to further impair PG recognition and cleavage by the catalytic domain. Truncation of the N-terminal domain by extracellular proteases thus ensures optimal activity of the enzyme once it has reached its substrate at the cell surface. N-terminal truncation of the N-terminal domain occurs during growth and can be detected by zymogram [8, 10]. It is tempting to assume that the proteolytic cleavage of the AtlA N-terminal domain is primarily mediated by the metalloprotease GelE, which has been associated with formation of short chains in *E*. *faecalis* [23]. However, the diplococcal state of *E*. *faecalis* is not restricted to strains producing GelE, indicating that other proteases can process AtlA. An example is *E*. *faecalis* JH2-2: although this strain is deficient for the production of GelE, it forms diplococci and very short chains.

Interestingly, the genes essential for AtlA glycosylation were previously shown to catalyze the production of diglucosyl-diacylglycerol [24]. This implies that (an)other glycosyl transferase(s) mediate(s) the direct glycosylation of AtlA. Further investigations are required to identify the corresponding enzyme(s). This task appears relatively difficult given the functional redundancy of glycosyl transferases. The *E*. *faecalis* V583 genome encodes 15 putative enzymes that could be responsible for AtlA glycosylation. Our results showed that in the absence of AtlA, the *gtfAB* deletion has no impact on septum cleavage. Altough we cannot formally rule out that glycosylation of other surface proteins can modulate cell separation, this effect (if any) is limited. The identification and characterization of GtfAB substrates awaits further analysis. Another open question deals with the degree of AtlA glycosylation. It is possible that the extent of AtlA glycosylation varies during growth or in response to environmental cues.

Recent studies have explored the impact of PG structure on substrate recognition and cleavage by the catalytic domain of PG hydrolases. The *N*-acetylglucosaminidase LytB, the *S*. *pneumoniae* functional homolog of *E*. *faecalis* AtlA, requires fully acetylated GlcNAc moieties for cleavage. A substrate-assisted catalytic mechanism involving anchimeric assistance by the C2-acetamido group of the GlcNAc moiety is likely to underpin this requirement [25]. Another example is the pneumococcal autolysin LytA, in which several amino acids in the vicinity of the catalytic residues contribute to positioning of the substrate in the catalytic cleft so that the scissile bond is at an optimal distance from the catalytic residue [26]. Our work revealed that the *N*-acetylglucosaminidase domain of AtlA is not essential for septum cleavage. However, swapping the AtlA catalytic domain for another domain results in a lower septum cleavage activity. One possibility is that in *E*. *faecalis*, AtlA has evolved to optimally recognize and cleave the local PG structure at the septum. Recent work in *E*. *coli* and *B*. *subtilis* suggested that septal PG is enriched in “denuded” glycan strands resulting from *N*-acetylmuramoyl-L-alanine amidase activity [27]. Whether denuded glycan strands represent an optimal substrate for AtlA remains to be tested. It is expected that the lack of peptide stems will increase the binding activity of the LysM domain [28]. Thus, measurements of the *N*-acetylglucosaminidase activity of AtlA against glycan chains and chains substituted by peptide stems should be carried out with the catalytic domain in isolation to uncouple binding of the AtlA enzyme to its substrate from catalysis.

Our previous work revealed that AtlA LysM motifs can fold independently and do not interact, thus suggesting that they behave as “beads on a string” [28]. This model implied that instead of forming a quaternary structure, LysM repeats bind PG in a cooperative manner. Here, we showed that septum cleavage increases with the number of LysM motifs, with the formation of diplococci requiring the presence of all six repeats. The model strain V583 encodes twelve proteins with LysM domains; four contain two repeats, seven contain a single repeat, AtlA being the only one with six repeats. Bearing in mind that the formation of diplococci or short chains by *E*. *faecalis* is critical for virulence, our results suggest that the lifestyle of this organism as a commensal has favored the emergence of multimodular LysM domains in AtlA.

During infection, the size of bacterial cells has a major impact on recognition by the immune system. One example is the cording morphology of mycobacteria, which correspond to snake-like structures formed by the end-to-end and side-to-side aggregation of bacilli [29]. The formation of large bacterial aggregates impairs phagocytosis and is required for virulence [30]. Another well-documented strategy to escape host immunity is the formation of filaments after invasion of epithelial cells by uropathogenic *E*. *coli* (UPEC) [31]. Inhibition of septation enhances resistance to phagocytosis and increase survival rates of UPEC and other pathogens in the host [32]. The exact mechanism by which filamentation inhibits uptake and killing by phagocytes is unclear. *In vitro* experiments using anisotropic polystyrene particles and alveolar macrophages revealed that the point of contact between phagocytes and particles is critical for phagocytosis initiation [33]. It has therefore been proposed that the increased cell length in filamentous bacteria reduces the probability of macrophages to encountering the cell poles that stimulate the formation of the phagocytic cup [34]. This early step in the uptake by immune cells appears to be a limiting factor, the internalization speed of filaments itself being similar to that of smaller particles [34]. In contrast, minimization of bacterial cell chains has been described as a strategy to overcome host immunity in *Streptococcus pneumoniae* [35]. The effect of cell chain length in *S*. *pneumoniae* involves subversion of complement-mediated opsonophagocytosis. Interestingly, our assay using monocyte-derived macrophages indicated that in the absence of complement, cell chains can be recognized and readily engulfed by phagocytes. Unlike UPEC filaments, the long chains of enterococci are pretty flexible and often form turns (S5B Fig). This is expected to generate a contact point with phagocytes that will favor cytoskeleton remodeling to form the phagocytic cup [34]. Impaired septum cleavage is primarily expected to restrict the capacity of the bacteria to disseminate and multiply in the host and is not expected to have any impact on clearance by phagocytes. In the context of a systemic infection in zebrafish larvae, diplococci are circulating in the bloodstream. The formation of long chains limits the dissemination of the bacteria, hence increasing their probability of encountering immune cells. We propose that the sequestration of *E*. *faecalis* inside the phagocytes prevents cell multiplication and release of the metalloprotease GelE that is essential to cause tissue damage and host death [17].

*E*. *faecalis* is a common nosocomial pathogen associated with a wide range of infections that can be life-threatening. It was recently shown to promote the growth of other microorganisms during polymicrobial infections [36]. This study suggests that targeting the enzymatic activity of AtlA, the autolysin dedicated to septum cleavage, represents a novel therapeutic strategy to eradicate *E*. *faecalis*.

## Methods

### Ethics statement

Monocyte-derived macrophages (MDM) were isolated from whole blood from healthy volunteers at the Sheffield Royal Hallamshire hospital with written informed consent prior to inclusion in the study, as approved by the South Sheffield Research Ethics Committee (07/Q2305/7) [37]. All samples were anonymised. Animal work was carried out according to guidelines and legislation set out in UK law in the Animals (Scientific Procedures) Act 1986 under Project License PPL 40/3574. Ethical approval was granted by the University of Sheffield Local Ethical Review Panel.

### Bacterial strains, plasmids and growth conditions

Bacterial strains and plasmids used in this study are described in Table 1. *E*. *coli* was grown at 37°C in Brain Heart Infusion (BHI) broth or agar 1.5% (w/v) supplemented with 200ug/ml erythromycin (for pGhost9 derivatives) and 100ug/ml ampicillin (pET derivatives). *E*. *faecalis* strains were grown in BHI broth or agar at 37°C, unless otherwise stated. When necessary, the medium was supplemented with 30 μg/ml erythromycin.

### Zebrafish strains and maintenance

London wild type (LWT) zebrafish [38] were provided by the aquarium facility at the University of Sheffield. Embryos were maintained in E3 medium at 28°C according to standard procedures previously described [39]. Phagocyte-depleted embryos were obtained following injection of phosphorodiamidate morpholino oligomers against *pu*.*1* as previously described [40].

### Microinjections of *E*. *faecalis* in zebrafish embryos

Cells were grown to mid-exponential phase (OD_600_~0.3) and harvested by centrifugation (5,000 x *g* for 10 min at room temperature). Bacteria were resuspended in filtered phosphate buffer saline (150 mM Na_2_HPO_4_, 20 mM KH_2_PO_4_, 150 mM NaCl [pH 7.5], PBS) and transferred to microcapillary pipettes. Embryos at 30 hours post fertilization (hpf) were anaesthetized, dechorionated, embedded in 3% (w/v) methylcellulose and injected individually with 2nl of a cell suspension corresponding to *ca*. 1,000 cells as previously described [40]. The number of cells injected was checked before and after each series of injections with a given strain. Zebrafish embryos were monitored at regular intervals until 90 h post infection (hpi).

### Immunostaining of phagocytes in zebrafish

Zebrafish larvae were fixed 1.5h post infection with 4% paraformaldehyde (m/v) at 4°C overnight and washed four times in PBS supplemented with 0.4% (v/v) TritonX 100 and 1% (v/v) DMSO (PBS-TxD). Samples were blocked in 5% (v/v) sheep serum in PBS-TxD for 1h at room temperature followed by one wash in PBS-TxD. Cells were incubated at 4°C overnight with primary antibodies (anti-L-plastin 1:400, gift from Paul Martin, University of Bristol). After four washes in blocking solution, embryos were incubated with secondary antibodies (goat anti-rabbit IgG, Alexa Fluor 647 conjugate, Life Technologies) for 2h at room temperature. Larvae were washed four times with PBS-TxD and fixed again with 4% paraformaldehyde (m/v) for 30 min at RT. Immunolabelled embryos were mounted using 0.8% (m/v) agarose in E3 medium and imaged with a confocal microscope.

### Imaging of infected larvae by confocal microscopy and quantification of uptake by phagocytes

Immunolabelled embryos were immersed in 0.8% (w/v) low melting point agarose in E3 medium and mounted flat on FluoroDish^™^ (World Precision Instruments Inc.). Images were collected using a DMi8 confocal microscope (Leica). Image acquisition was performed with the Volocity software and the images were processed with ImageJ 1.49v software. Bacterial phagocytosis was quantified using an ImageJ custom script called Fish Analysis v5 which can be obtained from http://sites.imagej.net/Willemsejj/ or via ImageJ updater. All bacteria were identified based on their (GFP, Channel 1) fluorescence. Subsequently, the fluorescence intensity of the phagocytes (Alexa 647, Channel 2) surrounding the phagocytosed bacteria was measured. The phagocytosed bacteria had high fluorescence intensity of Channel 2 and the cut off of 2 was used to discriminate the phagocytosed from non-phagocytosed bacteria. The area of phagocytosed bacteria was compared with the area of non-phagocytosed bacteria and their ratio was calculated.

### Macrophage isolation and culture

Monocyte-derived macrophages (MDM) were isolated from whole blood from healthy volunteers. Peripheral blood mononuclear cells were isolated by Ficoll Plaque (GE Healthcare) density centrifugation. To differentiate PBMC into monocyte-MDM 2×10^6^ PBMC/ml were plated in RPMI 1640 media (Lonza) with 2 mmol/l L-glutamine (Gibco BRL) containing 10% human AB serum (First Link (UK) LTD) in 24-well plates (Costar). After 24h, non-adherent cells were removed, and adherent cells were cultured in RPMI with 10% heat-treated fetal bovine serum (FBS; Lonza) in 5% CO_2_ at 37°C to give a final concentration of approximately 2×10^5^ MDM/ml at day 14 [37].

### *In vitro* phagocytosis assay

*E*. *faecalis* strains were grown to OD_600_ = 0.6 and stored in frozen aliquots at -80°C. Viable counts were determined upon thawing and used to calculate volumes necessary to give desired multiplicity of infection (MOI). Bacteria were labelled with pHrodo dye (pHrodo Red, succinimidyl ester, Invitrogen) as previously described [41]. Briefly, bacteria were washed in phosphate-buffered saline before being incubated with 10.2 μM pHrodo in 0.1M sodium bicarbonate pH8.3 for 30min at 37°C protected from light. Excess dye was washed off before MDM were challenged with pHrodo labelled bacteria at MOI = 100 for 4 hours at 37°C. Cells were then fixed in 2% paraformaldehyde and fluorescence (Ex/Em 560/585nm) measured on a Varioskan Flash multimode reader (Thermo Scientific). Relative fluorescence values (RFU) of cell only wells were subtracted from readings to control for autofluorescence.

### Construction of pGhost derivatives for allele replacement

A similar strategy was followed to construct all plasmids for allele replacement except pGABhis, for which the whole insert was synthesized and cloned into pGhost9 using XhoI and EcoRI restriction sites. For pGAAhis, pGBBhis, pGDN, pGtfAB, pGAtlA1, pGAtlA1-2, pGAtlA1-3, pGAtlA1-4, pGAtlA1-5, two homology regions were amplified and fused by overlap extension using PCR [42]. A 5’ homology region (referred to as H1) was amplified with oligonucleotides H11 (sense) and H12 (antisense). It was fused to a 3’ homology region (referred to as H2) amplified with oligonucleotides H21 (sense) and H22 (antisense). The assembled PCR fragment flanked by two restriction sites was digested and cloned into pGhost9 similarly digested. Oligonucleotide sequences and restriction sites used for cloning are described in S1 Table.

For pGAtlA*, three homology regions (H1, H2, H3) were fused by overlap extension. The resulting plasmid contains a catalytic domain flanked by NcoI and BamHI sites. The NcoI-BamHI fragment encoding the *N*-acetylglucosaminidase activity of AtlA was swapped for NcoI-BamHI fragments encoding catalytic domains with distinct catalytic activities to produce pGAtlA-Cse, pGAtlA-AtlB and pGAtlA-Ami generated by PCR (see Supplemental Experimental Procedures for oligonucleotide sequences). The sequences of the chimeric proteins encoded by these plasmids are described in S3 Fig.

### Construction of *E*. *faecalis* mutants

Isogenic derivatives of *E*. *faecalis* JH2-2 were constructed by allele exchange using the procedure previously described [8]. Briefly, pGhost derivatives were electroporated into JH2-2 and transformants were selected at a permissive temperature (28°C) on BHI plates with erythromycin. To induce single crossover recombination, transformants were grown at a non-permissive temperature (42°C) in the presence of erythromycin. The second recombination event leading to plasmid excision was obtained after 5 serial subcultures at 28°C without erythromycin. The last overnight subculture was plated at 42°C without erythromycin. A clone harboring a double crossover mutation was identified by PCR and Southern blot hybridization.

To construct double mutants JH2-2 *atlA*_*1-4*ΔN_ and JH2-2 *atlA*_*1-4*_Δ*gtfAB*, the deletion of two LysM repeats was introduced in JH2-2 *atlA*_ΔN_ and JH2-2 Δ*gtfAB* backgrounds using the pG*atlA*_1-4_ plasmid (Table 1).

**Table 1:** Bacterial strains and plasmids used in this study.

Strains, plasmids	Relevant properties or genotype^a^	Source or reference
**Strains**		
***Enterococcus faecalis***		
OG1RF	Plasmid-free, virulent laboratory strain isolated from the oral cavity	[43]
OG1RF *gfp*	OG1RF producing the GFP encoded by pMV158	[17]
OG1RFΔ*atlA*	OG1RF mutant harboring a deletion in *atlA*	This work
OG1RFΔ*atlAgfp*	OG1RF Δ*atlA* derivative producing the GFP encoded by pMV158	This work
JH2-2	Plasmid-free laboratory strain	[44]
Δ*atlA*	JH2-2 mutant harboring an in-frame deletion of *atlA*	[8]
P*atlA*::*atlB-his*	JH2-2 producing a C-terminally his-tagged AtlB under the *atlA* promoter; In this strain, the *atlA*^-^ open reading frame is replaced by that of *atlB*	This work
P*atlA*::*atlA-his*	JH2-2 producing a C-terminally his-tagged AtlA (native locus)	This work
P*atlB*::*atlB-his*	JH2-2 producing a C-terminally his-tagged AtlB (native locus)	This work
*atlA*_ΔN_	JH2-2 producing AtlA without its N-terminal domain	This work
*atlA*_1-4ΔN_	JH2-2 *atlA*_ΔN_ derivative producing AtlA lacking 2 C-terminal LysM repeats	This work
Δ*gtfAB*	JH2-2 derivative with an in-frame deletion of the *gtfAB* operon	This work
Δ*atlA*Δ*gtfAB*	JH2-2 Δ*atlA* derivative with an in-frame deletion of the *gtfAB* operon	This work
*atlA*_1-4_Δ*gtfAB*	JH2-2 *gtfAB* derivative producing AtlA lacking 2 C-terminal LysM repeats	This work
atlA*	JH2-2 producing AtlA with a catalytic domain flanked by NcoI and BamHI sites	This work
*atlA*_Cse_	JH2-2 atlA* derivative producing AtlA with endopeptidase activity	This work
*atlA*_AtlB_	JH2-2 atlA* derivative producing AtlA with *N*-acetylmuramidase activity	This work
*atlA*_Ami_	JH2-2 atlA* derivative producing AtlA with amidase activity	This work
*atlA*_1-5_	JH2-2 producing AtlA lacking the last C-terminal LysM repeat	This work
*atlA*_1-4_	JH2-2 producing AtlA lacking the last 2 C-terminal LysM repeats	This work
*atlA*_1-3_	JH2-2 producing AtlA lacking the last 3 C-terminal LysM repeats	This work
*atlA*_1-2_	JH2-2 producing AtlA lacking the last 4 C-terminal LysM repeats	This work
*atlA*_1_	JH2-2 producing AtlA lacking the last 5 C-terminal LysM repeats	This work
***Escherichia coli***		
TG1	Host for plasmid propagation	Lab stock
TG1(RepA)	TG1 derivative producing RepA for pGhost propagation at 37°C	P. Serror
BL21(DE3)	BL21 derivative for protein expression	Novagen
**Plasmids**		
pGhost9	Thermosensitive plasmid for gene replacement in *E*. *faecalis* (Erm^R^)	[45]
pMV158	Replicative plasmid for constitutive *gfp* expression	[46]
pGHH0799	pGhost9 derivative used to construct strain OG1RF Δ*atlA*	[8]
pGABhis	pGhost9 derivative used to construct strain P*atlA*::*atlB-his*	This work
pGAAhis	pGhost9 derivative used to construct strain P*atlA*::*atlA-his*	This work
pGBBhis	pGhost9 derivative used to construct strain P*atlB*::*atlB-his*	This work
pGDN	pGhost9 derivative used to construct strain *atlA*_ΔN_	This work
pGgtfAB	pGhost9 derivative used to construct strain Δ*gtfAB*	This work
pGatlA1-5	pGhost9 derivative used to construct strain *atlA*_1-5_	This work
pGatlA1-4	pGhost9 derivative used to construct strain *atlA*_1-4_	This work
pGatlA1-3	pGhost9 derivative used to construct strain *atlA*_1-3_	This work
pGatlA1-2	pGhost9 derivative used to construct strain *atlA*_1-2_	This work
pGatlA1	pGhost9 derivative used to construct strain *atlA*_1_	This work
pGatlA*	pGhost9 derivative used to construct strain atlA*	This work
pGatlA-Cse	pGhost9 derivative used to construct strain *atlA*_Cse_	This work
pGatlA-AtlB	pGhost9 derivative used to construct strain *atlA*_AtlB_	This work
pGatlA-Ami	pGhost9 derivative used construct strain *atlA*_Ami_	This work
pET2818	pET28a derivative for overexpression of His-tagged proteins (Amp^R^)	[10]
pET-AtlA_TEV_	pET2818 encoding AtlA with a TEV site upstream of the catalytic domain	This work
pET-AtlB	pET2818 encoding AtlB	This work
pET-AtlAB	pET2818 encoding AtlA with a LysM domain replaced by that of AtlB	This work
pET-AtlBA	pET2818 encoding AtlB with a LysM domain replaced by that of AtlA	This work

^a^ Amp^R^, resistant to ampicillin; Erm^R^, resistant to erythromycin

### Construction of pET derivatives for protein expression in *E*. *coli*

pET2818 was used as an expression vector to produce C-terminally His-tagged recombinant proteins. To construct pET-AtlA_TEV_, a cleavage site recognized by the Tobacco Etched Virus (TEV) protease was introduced by PCR by fusing two amplified products (named H1 and H2). For pET-AtlAB and pET-AtlBA, a DNA fragment encoding the N-terminal domain of AtlA or AtlB (referred to as H1) was fused to a DNA fragment encoding the LysM domain of AtlA or AtlB (referred to as H2). The resulting fragments were cut by NcoI and BamHI and cloned into pET2818 that had been similarly digested. Specific oligonucleotides used for each construct are described in S1 Table. The sequences of recombinant proteins expressed in *E*. *coli* are described in S3 Fig.

### Production and purification of his-tagged recombinant proteins produced in *E*. *coli*

*E*. *coli* BL21(DE3) cells harboring pET-derivatives were grown to an optical density at 600 nm (OD_600_) of 0.7 and production of recombinant proteins was induced by addition of 1 mM isopropyl-β-D-thiogalactopyranoside. The cells were harvested 4h after induction, resuspended in buffer A (50 mM Tris-HCl [pH 8.0] containing 300 mM NaCl) and sonicated (5 times 30 sec at 20% output using a Branson Sonifier 450). Soluble proteins were recovered after centrifugation (45,000 x *g*, 20 min at 4°C), loaded onto Ni^2+^-nitrilotriacetate agarose resin (Qiagen GmbH, Hilden, Germany), washed with 10 mM imidazole in buffer A and eluted with 300 mM imidazole in buffer A. Recombinant his-tagged proteins were further purified by size exclusion chromatography on a Superdex75 HR 26/60 column (Amersham biosciences, Uppsala, Sweden) equilibrated with PBS. The fractions were analyzed by SDS-PAGE and pooled. Protein concentration was estimated by measuring the absorbance, using a theoretical extinction coefficient at 280 nm (http://www.expasy.org). Proteins were kept frozen at -80°C in PBS supplemented with 25% glycerol. AtlB stocks were available from previous studies [8].

### Protein preparation from *E*. *faecalis* cultures

Proteins from supernatants were prepared from exponentially growing cultures (OD_600_~0.4). Supernatants were precipitated by addition of TCA (10% v/v final). After 10 min on ice, proteins were recovered by centrifugation (15,000 x *g*, 10 min at room temperature), washed in 100% acetone, dried and resuspended in SDS-PAGE loading buffer (1ml/equivalent OD_600_ = 50).

For the detection of His-tagged AtlA and AtlB produced under the control of the *atlA* promoter, proteins were prepared from cultures in late exponential phase (OD_600_~1). One ml of culture (containing both cells and supernatant) was transferred to a tube containing 250μL of glass beads (100μm in diameter, Sigma). Cells were mechanically disrupted using a FastPrep device (six cycles of 40 sec at maximum speed with 5 min pauses between cycles). Loading buffer was added to the protein samples and equivalents of 40, 20 and 10 μl of the cultures were separated on a 10% SDS-PAGE.

### Western blot detection of AtlA and AtlB

Proteins were transferred to a nitrocellulose membrane. After a blocking step for 1h at room temperature in Tris buffer saline (TBS, 10mM Tris-HCl pH7.4, 150mM NaCl) supplemented with tween-20 (0.025%, v/v) and milk (2%, m/v), the membrane was incubated with rabbit polyclonal anti-AtlA antibodies raised against the catalytic domain of AtlA (1:1,000 dilution) or polyclonal anti-His antibodies (1:2,000 dilution; Ebioscience). Proteins were detected using goat polyclonal anti-rabbit antibodies conjugated to horseradish peroxidase (Sigma) at a dilution of 1:20,000 and clarity Western ECL Blotting Substrate (BioRad).

### Detection of AtlA activity by zymogram

Proteins from the supernatant were separated on a 10% SDS-PAGE containing *Micrococcus luteus* autoclaved cells as a substrate (final OD_600_ = 2). After electrophoresis the gel was rinsed in distilled water and proteins were renatured at 37°C in a buffer containing 50mM Tris-HCl (pH7.5) and 0.1% (v/v TritonX-100).

### Flow cytometry analysis of cell chain length

Cells were grown overnight without agitation at 37°C. Cells were diluted 1:100 into fresh broth (OD_600_ ~0.02) and grown in standing cultures to mid-exponential phase (OD_600_~0.2 to 0.4). Bacteria were diluted 1:100 in phosphate buffer saline filtered on a 0.22μm pore size membrane (Millex-GV syringe filter unit, Millipore) to eliminate salt crystals which could interfere with measurements and analyzed by flow cytometry using Millipore Guava Easy Cyte system. Light scatter data were obtained with logarithmic amplifiers for 20,000 events.

To measure the septum cleavage activity of recombinant proteins, the OG1RF Δ*atlA* mutant was grown to exponential phase (OD_600_ = 0.2), collected by centrifugation, and bacterial chains were resuspended in filtered PBS containing various concentrations of recombinant proteins. Cell size distribution was determined by flow cytometry after 15 min of incubation at 37°C. Relative logs of forward scattered light values (FS log) were collected for 5,000 events and expressed as a percentage of the control strain incubated in the absence of enzyme.

### Light and fluorescent microscopy analysis of bacteria

Cells were grown to mid-exponential phase (final OD_600_~0.3) and fixed with 1.6% paraformaldehyde in PBS for 30min at RT. After fixation, bacteria were washed twice in distilled water and mounted onto poly-L-lysine coated slides and imaged using a DeltaVision deconvolution microscope equipped with an UplanSApo 100x oil (NA 1.4) objective and a Photometrics Coolsnap HQ CCD camera. ImageJ software was used to optimize contrast and to count the numbers of cells per chain.

### Statistical analyses

All experiments reported in this study correspond to at least three biological replicates. Statistical analyses were performed using GraphPad Prism version 6.0e. Comparisons between survival curves were made using the log rank (Mantel-Cox) test. Median FSC values were compared using a two-tailed, unpaired Student’s t test with Welch’s correction. Comparison of OG1RF and Δ*atlA* derivative uptake *in vitro* by MDM was carried out using a paired Student’s t test. Comparison of uptake by zebrafish macrophages was carried out using an unpaired non-parametric Dunn’s multiple comparisons test. The number of cells per chain was compared using a non-parametric Mann-Whitney *U* test. Statistical significance was assumed at *P* values below 0.05.

## Supporting information

S1 Fig Analysis of *E*. *faecalis* strains producing an N-terminally truncated AtlA. **A**. Western blot detection of AtlA proteins in culture supernatants. Supernatant proteins from exponentially growing cells were recovered by centrifugation, precipitated with 10% (m/v) TCA, washed with acetone and resuspended in PBS. Following SDS-PAGE and transfer on a nitrocellulose membrane, AtlA proteins were detected using an anti-AtlA polyclonal serum against the catalytic domain of AtlA. WT, *E*. *faecalis* JH2-2; *atlA*_ΔN,_ derivative expressing AtlA truncated from the N-terminal domain; *atlA*_1-4_, derivative expressing AtlA truncated from the two C-terminal LysM modules; AtlA_1-4ΔN_, AtlA_1-4_ truncated from the N-terminal domain WT; A strain with an in-frame deletion of *atlA* (Δ*atlA*) was used as a negative control. **B**. Average numbers of cells per chain formed by WT (3.0 ± 1.6; n = 427 cells); *atlA*_ΔN_ (2.6 ± 1.4; n = 534 cells); *atlA*_1-4_ (9.4 ± 4.8; n = 442 cells) and *atlA*_1-4ΔN_ (5.9 ± 3.4; n = 610 cells) strains; ****P<0.0001. **C**. Light microscopy images showing cell chain lengths of the mutants. **D**. Sequence of AtlA N-terminal domain (residues 54 to 172). S/T/E residues are indicated in red.
(TIF)

S2 Fig Analysis of *E*. *faecalis* strains lacking AtlA glycosylation. **A**. Western blot detection of AtlA proteins in culture supernatants (as described in S1 Fig). WT, *E*. *faecalis* JH2-2; *atlA*_1-4_, derivative expressing AtlA truncated from the two C-terminal LysM modules; Δ*gtfAB*, derivative with an in-frame deletion of the Δ*gtfAB* operon; *atlA*_1-4_Δ*gtfAB*, Δ*gtfAB* derivative with the truncation of the two C-terminal LysM modules of *atlA*. A strain with an in-frame deletion of *atlA*, Δ*atlA* was used as a negative control. **B**. Average numbers of cells per chain formed by WT (3.0 ± 1.6; n = 427 cells); Δ*gtfAB* (3.0 ± 1.3; n = 364 cells); *atlA*_1-4_ (9.4 ± 4.8; n = 442 cells); Δ*gtfAB atlA*_1-4_ (6.5 ± 3.7; n = 298 cells); NS, *P*>0.05; ***, *P* = 0.0005. **C**. Light microscopy images showing cell chain lengths of the mutants.
(TIF)

S3 Fig Description of AtlA derivatives expressed in *E*. *faecalis* and *E*. *coli*. **A**. Schematic representation of the amino acid modifications introduced on either side of the catalytic domain for cloning purposes. **B**. Sequence of the AtlA variants with a swapped catalytic domain. Sequences in red correspond to catalytic domains of chimeric proteins expressed by recombinant *E*. *faecalis* strains analyzed in Fig 6. **C**. Western blot detection of chimeric proteins. Protein samples corresponding to crude extracts were run on an SDS-PAGE, transferred on a nitrocellulose membrane and probed with an anti-LysM polyclonal serum. The arrowheads indicate unspecific signals. **D**. Sequences in blue correspond to LysM domains used to construct the chimeric recombinant proteins expressed in *E*. *coli* (see Fig 5).
(TIF)

S4 Fig Characterization of *E*. *faecalis* strains producing a LysM domain with a variable number of LysM repeats. **A**. Western blot detection of AtlA proteins in culture supernatants. Cells were grown until exponential phase (OD_600_ = 0.2–0.5) and spun down. Supernatants were precipitated with 10% (m/v) TCA prior to detection of AtlA as described in supplementary Fig 1. Bands with the expected molecular weights were detected in all the strains. **B**. Zymogram analysis of AtlA activity in culture supernatants. Samples analyzed in (**A**) were loaded on an SDS-PAGE containing autoclaved *M*. *luteus* cells (OD_600_ = 2). After migration, the gel was rinsed and incubated in renaturing buffer to detect AtlA activity. Truncation of LysM repeats was associated with a decrease in AtlA activity. **C**. Light microscopy images showing cell chain lengths of the mutants.
(TIF)

S5 Fig Analysis of the virulence of *E*. *faecalis* Δ*atlA* mutants forming long chains. **A**. Comparison of median forward scattered (FSC) light values corresponding to the cell chain lengths of WT (OG1RF), Δ*atlA* and sonicated Δ*atlA* (Δ*atlA*^S^) strains. **B**. Light microscopy images showing cell chain lengths of WT and Δ*atlA* derivatives expressing cytoplasmic GFP. **C**. Survival of zebrafish larvae (n>20) following infection with *E*. *faecalis* OG1RF (WT) and *atlA* isogenic deletion mutant before (Δ*atlA*) and after (Δ*atlA*^S^) sonication to disperse long chains. The results corresponding to three independent experiments are shown. For each experiment, the number of cells injected (determined after sonication) is indicated. **D**. *P* values resulting from pairwise comparisons using the log rank test. **E**. survival of phagocyte-depleted zebrafish larvae following injection with 1250 cells of *E*. *faecalis* OG1RF (WT) or Δ*atlA*.
(TIF)

S6 Fig Analysis of the impact of mild sonication on *E*. *faecalis* OG1RF virulence. **A**. Survival of zebrafish larvae (n>20) following infection with *E*. *faecalis* OG1RF (WT) and OG1RF sonicated (WT^S^) cells. The results corresponding to three independent experiments are shown. For each experiment, the number of cells injected (determined after sonication) is indicated. **B**. *P* values resulting from pairwise comparisons using the log rank test.
(TIF)

S7 Fig Analysis of bacterial growth rates of *E*. *faecalis* Δ*atlA* and Δ*atlA*^S^. Cells from an overnight culture in BHI were diluted to an OD_600_ of 0.01 in 25ml BHI and growth of standing cultures were monitored over 7 hours. The growth rate of each strain was determined using the OD values between 60 and 240 minutes (exponential growth). The data presented are the average of 3 independent cultures. OD values of individual growth curves are presented.
(TIF)

S1 Table Oligonucleotides used in this study. (DOC)

## References

[1] Arias CA, Murray BE. The rise of the *Enterococcus*: beyond vancomycin resistance. Nature reviews Microbiology. 2012;10(4):266–78. Epub 2012/03/17. doi: 10.1038/nrmicro2761 22421879 10.1038/nrmicro2761 PMC3621121

[2] Bradley CR, Fraise AP. Heat and chemical resistance of enterococci. J Hosp Infect. 1996;34(3):191–6. 8923273 10.1016/s0195-6701(96)90065-1

[3] Chang S, Sievert DM, Hageman JC, Boulton ML, Tenover FC, Downes FP, et al Infection with vancomycin-resistant *Staphylococcus aureus* containing the *vanA* resistance gene. The New England journal of medicine. 2003;348(14):1342–7. Epub 2003/04/04. doi: 10.1056/NEJMoa025025 12672861 10.1056/NEJMoa025025

[4] Noble WC, Virani Z, Cree RG. Co-transfer of vancomycin and other resistance genes from *Enterococcus faecalis* NCTC 12201 to *Staphylococcus aureus*. FEMS microbiology letters. 1992;72(2):195–8. Epub 1992/06/01. 1505742 10.1016/0378-1097(92)90528-v

[5] Sievert DM, Ricks P, Edwards JR, Schneider A, Patel J, Srinivasan A, et al Antimicrobial-resistant pathogens associated with healthcare-associated infections: summary of data reported to the National Healthcare Safety Network at the Centers for Disease Control and Prevention, 2009–2010. Infection control and hospital epidemiology. 2013;34(1):1–14. Epub 2012/12/12. doi: 10.1086/668770 23221186 10.1086/668770

[6] de Been M, Pinholt M, Top J, Bletz S, Mellmann A, van Schaik W, et al Core genome multilocus sequence typing scheme for high-resolution typing of *Enterococcus faecium*. Journal of clinical microbiology. 2015;53(12):3788–97. Epub 2015/09/25. doi: 10.1128/JCM.01946-15 26400782 10.1128/JCM.01946-15 PMC4652124

[7] Guzman Prieto AM, van Schaik W, Rogers MR, Coque TM, Baquero F, Corander J, et al Global emergence and dissemination of Enterococci as nosocomial pathogens: attack of the clones? Frontiers in microbiology. 2016;7:788 Epub 2016/06/16. doi: 10.3389/fmicb.2016.00788 27303380 10.3389/fmicb.2016.00788 PMC4880559

[8] Mesnage S, Chau F, Dubost L, Arthur M. Role of *N*-acetylglucosaminidase and *N*-acetylmuramidase activities in *Enterococcus faecalis* peptidoglycan metabolism. The Journal of biological chemistry. 2008;283(28):19845–53. Epub 2008/05/21. doi: 10.1074/jbc.M802323200 18490448 10.1074/jbc.M802323200

[9] Emirian A, Fromentin S, Eckert C, Chau F, Dubost L, Delepierre M, et al Impact of peptidoglycan *O*-acetylation on autolytic activities of the *Enterococcus faecalis N*-acetylglucosaminidase AtlA and *N*-acetylmuramidase AtlB. FEBS Lett. 2009;583:3033–8. Epub 2009/08/19. doi: 10.1016/j.febslet.2009.08.010 19686739 10.1016/j.febslet.2009.08.010

[10] Eckert C, Lecerf M, Dubost L, Arthur M, Mesnage S. Functional analysis of AtlA, the major *N*-acetylglucosaminidase of *Enterococcus faecalis*. Journal of bacteriology. 2006;188(24):8513–9. doi: 10.1128/JB.01145-06 17041059 10.1128/JB.01145-06 PMC1698247

[11] Lee IC, van S, II, Tomita S, Morsomme P, Rolain T, Hols P, et al GtfA and GtfB are both required for protein *O*-glycosylation in *Lactobacillus plantarum*. Journal of bacteriology. 2014;196(9):1671–82. Epub 2014/02/18. doi: 10.1128/JB.01401-13 24532775 10.1128/JB.01401-13 PMC3993320

[12] Li Y, Huang X, Li J, Zeng J, Zhu F, Fan W, et al Both GtfA and GtfB are required for SraP glycosylation in *Staphylococcus aureus*. Current microbiology. 2014;69(2):121–6. Epub 2014/03/25. doi: 10.1007/s00284-014-0563-2 24658735 10.1007/s00284-014-0563-2

[13] Wu R, Zhou M, Wu H. Purification and characterization of an active *N*-acetylglucosaminyltransferase enzyme complex from Streptococci. Applied and environmental microbiology. 2010;76(24):7966–71. Epub 2010/10/26. doi: 10.1128/AEM.01434-10 20971868 10.1128/AEM.01434-10 PMC3008268

[14] Reste de Roca F, Duche C, Dong S, Rince A, Dubost L, Pritchard DG, et al Cleavage specificity of *Enterococcus faecalis* EnpA (EF1473), a peptidoglycan endopeptidase related to the LytM/lysostaphin family of metallopeptidases. Journal of molecular biology. 2010;398(4):507–17. Epub 2010/03/30. doi: 10.1016/j.jmb.2010.03.033 20347848 10.1016/j.jmb.2010.03.033

[15] Oshida T, Sugai M, Komatsuzawa H, Hong YM, Suginaka H, Tomasz A. A *Staphylococcus aureus* autolysin that has an *N*-acetylmuramoyl-L-alanine amidase domain and an endo-beta-*N*-acetylglucosaminidase domain: cloning, sequence analysis, and characterization. Proc Natl Acad Sci U S A. 1995;92(1):285–9. 7816834 10.1073/pnas.92.1.285 PMC42863

[16] Layec S, Gerard J, Legue V, Chapot-Chartier MP, Courtin P, Borges F, et al The CHAP domain of Cse functions as an endopeptidase that acts at mature septa to promote Streptococcus thermophilus cell separation. Molecular microbiology. 2009;71(5):1205–17. Epub 2009/01/28. doi: 10.1111/j.1365-2958.2009.06595.x 19170887 10.1111/j.1365-2958.2009.06595.x

[17] Prajsnar TK, Renshaw SA, Ogryzko NV, Foster SJ, Serror P, Mesnage S. Zebrafish as a novel vertebrate model to dissect enterococcal pathogenesis. Infection and immunity. 2013;81(11):4271–9. Epub 2013/09/05. doi: 10.1128/IAI.00976-13 24002065 10.1128/IAI.00976-13 PMC3811811

[18] Dubee V, Chau F, Arthur M, Garry L, Benadda S, Mesnage S, et al The *in vitro* contribution of autolysins to bacterial killing elicited by amoxicillin increases with inoculum size in *Enterococcus faecalis*. Antimicrobial agents and chemotherapy. 2011;55(2):910–2. Epub 2010/11/26. doi: 10.1128/AAC.01230-10 21098238 10.1128/AAC.01230-10 PMC3028817

[19] Ahn SJ, Burne RA. The atlA operon of Streptococcus mutans: role in autolysin maturation and cell surface biogenesis. Journal of bacteriology. 2006;188(19):6877–88. doi: 10.1128/JB.00536-06 16980491 10.1128/JB.00536-06 PMC1595523

[20] Thomas V, Hiromasa Y, Harms N, Thurlow L, Tomich J, Hancock LE. A fratricidal mechanism is responsible for eDNA release and contributes to biofilm development of *Enterococcus faecalis*. Molecular microbiology. 2009;72(4):1022–36. Epub 2009/04/21. doi: 10.1111/j.1365-2958.2009.06703.x 19400795 10.1111/j.1365-2958.2009.06703.x PMC2779696

[21] Miksa M, Komura H, Wu R, Shah KG, Wang P. A novel method to determine the engulfment of apoptotic cells by macrophages using pHrodo succinimidyl ester. Journal of immunological methods. 2009;342(1–2):71–7. Epub 2009/01/13. doi: 10.1016/j.jim.2008.11.019 19135446 10.1016/j.jim.2008.11.019 PMC2675277

[22] Thiercelin ME. Sur un diplococque saprophyte de l'intestin susceptible de devenir pathogene. CR Soc Biol. 1899;5:269–71.

[23] Waters CM, Antiporta MH, Murray BE, Dunny GM. Role of the *Enterococcus faecalis* GelE protease in determination of cellular chain length, supernatant pheromone levels, and degradation of fibrin and misfolded surface proteins. Journal of bacteriology. 2003;185(12):3613–23. doi: 10.1128/JB.185.12.3613-3623.2003 12775699 10.1128/JB.185.12.3613-3623.2003 PMC156229

[24] Theilacker C, Sanchez-Carballo P, Toma I, Fabretti F, Sava I, Kropec A, et al Glycolipids are involved in biofilm accumulation and prolonged bacteraemia in *Enterococcus faecalis*. Molecular microbiology. 2009;71(4):1055–69. Epub 2009/01/28. 19170884 10.1111/j.1365-2958.2008.06587.x

[25] Rico-Lastres P, Diez-Martinez R, Iglesias-Bexiga M, Bustamante N, Aldridge C, Hesek D, et al Substrate recognition and catalysis by LytB, a pneumococcal peptidoglycan hydrolase involved in virulence. Scientific reports. 2015;5:16198 Epub 2015/11/06. doi: 10.1038/srep16198 26537571 10.1038/srep16198 PMC4633669

[26] Sandalova T, Lee M, Henriques-Normark B, Hesek D, Mobashery S, Mellroth P, et al The crystal structure of the major pneumococcal autolysin LytA in complex with a large peptidoglycan fragment reveals the pivotal role of glycans for lytic activity. Molecular microbiology. 2016;101(6):954–67. Epub 2016/06/09. doi: 10.1111/mmi.13435 27273793 10.1111/mmi.13435 PMC5014641

[27] Yahashiri A, Jorgenson MA, Weiss DS. Bacterial SPOR domains are recruited to septal peptidoglycan by binding to glycan strands that lack stem peptides. Proc Natl Acad Sci U S A. 2015;112(36):11347–1152. Epub 2015/08/26. doi: 10.1073/pnas.1508536112 26305949 10.1073/pnas.1508536112 PMC4568695

[28] Mesnage S, Dellarole M, Baxter NJ, Rouget JB, Dimitrov JD, Wang N, et al Molecular basis for bacterial peptidoglycan recognition by LysM domains. Nature communications. 2014;5:4269 Epub 2014/07/01. doi: 10.1038/ncomms5269 24978025 10.1038/ncomms5269 PMC4083421

[29] Julian E, Roldan M, Sanchez-Chardi A, Astola O, Agusti G, Luquin M. Microscopic cords, a virulence-related characteristic of *Mycobacterium tuberculosis*, are also present in nonpathogenic mycobacteria. Journal of bacteriology. 2010;192(7):1751–60. Epub 2010/01/26. doi: 10.1128/JB.01485-09 20097851 10.1128/JB.01485-09 PMC2838037

[30] Bernut A, Lutfalla G, Kremer L. [Looking through zebrafish to study host-pathogen interactions]. Medecine sciences: M/S. 2015;31(6–7):638–46. Epub 2015/07/15. doi: 10.1051/medsci/20153106017 26152168 10.1051/medsci/20153106017

[31] Justice SS, Harrison A, Becknell B, Mason KM. Bacterial differentiation, development, and disease: mechanisms for survival. FEMS microbiology letters. 2014;360(1):1–8. Epub 2014/09/18. doi: 10.1111/1574-6968.12602 25228010 10.1111/1574-6968.12602 PMC4227932

[32] Justice SS, Hunstad DA, Cegelski L, Hultgren SJ. Morphological plasticity as a bacterial survival strategy. Nature reviews Microbiology. 2008;6(2):162–8. Epub 2007/12/25. doi: 10.1038/nrmicro1820 18157153 10.1038/nrmicro1820

[33] Champion JA, Mitragotri S. Role of target geometry in phagocytosis. Proc Natl Acad Sci U S A. 2006;103(13):4930–4. Epub 2006/03/22. doi: 10.1073/pnas.0600997103 16549762 10.1073/pnas.0600997103 PMC1458772

[34] Möller J, Luehmann T, Hall H, Vogel V. The race to the pole: how high-aspect ratio shape and heterogeneous environments limit phagocytosis of filamentous *Escherichia coli* bacteria by macrophages. Nano letters. 2012;12(6):2901–5. Epub 2012/05/18. doi: 10.1021/nl3004896 22591454 10.1021/nl3004896

[35] Dalia AB, Weiser JN. Minimization of bacterial size allows for complement evasion and is overcome by the agglutinating effect of antibody. Cell host & microbe. 2011;10(5):486–96. Epub 2011/11/22. doi: 10.1016/j.chom.2011.09.009 22100164 10.1016/j.chom.2011.09.009 PMC3222866

[36] Keogh D, Tay WH, Ho YY, Dale JL, Chen S, Umashankar S, et al Enterococcal metabolite cues facilitate interspecies niche modulation and polymicrobial infection. Cell host & microbe. 2016;20(4):493–503. Epub 2016/10/14. doi: 10.1016/j.chom.2016.09.004 27736645 10.1016/j.chom.2016.09.004 PMC5076562

[37] Dockrell DH, Lee M, Lynch DH, Read RC. Immune-mediated phagocytosis and killing of *Streptococcus pneumoniae* are associated with direct and bystander macrophage apoptosis. The Journal of infectious diseases. 2001;184(6):713–22. Epub 2001/08/23. doi: 10.1086/323084 11517432 10.1086/323084

[38] Bojarczuk A, Miller KA, Hotham R, Lewis A, Ogryzko NV, Kamuyango AA, et al *Cryptococcus neoformans* intracellular proliferation and capsule size determines early macrophage control of infection. Scientific reports. 2016;6:21489 Epub 2016/02/19. doi: 10.1038/srep21489 26887656 10.1038/srep21489 PMC4757829

[39] Nusslein-Volhard C. Zebrafish A practical approach. New York, NY: Oxford Universty Press; 2002.

[40] Prajsnar TK, Cunliffe VT, Foster SJ, Renshaw SA. A novel vertebrate model of *Staphylococcus aureus* infection reveals phagocyte-dependent resistance of zebrafish to non-host specialized pathogens. Cellular microbiology. 2008;10(11):2312–25. Epub 2008/08/22. doi: 10.1111/j.1462-5822.2008.01213.x 18715285 10.1111/j.1462-5822.2008.01213.x

[41] Jubrail J, Morris P, Bewley MA, Stoneham S, Johnston SA, Foster SJ, et al Inability to sustain intraphagolysosomal killing of *Staphylococcus aureus* predisposes to bacterial persistence in macrophages. Cellular microbiology. 2016;18(1):80–96. Epub 2015/08/08. doi: 10.1111/cmi.12485 26248337 10.1111/cmi.12485 PMC4778410

[42] Ho SN, Hunt HD, Horton RM, Pullen JK, Pease LR. Site-directed mutagenesis by overlap extension using the polymerase chain reaction. Gene. 1989;77(1):51–9. Epub 1989/04/15. 2744487 10.1016/0378-1119(89)90358-2

[43] Dunny GM, Brown BL, Clewell DB. Induced cell aggregation and mating in *Streptococcus faecalis*: evidence for a bacterial sex pheromone. Proc Natl Acad Sci U S A. 1978;75(7):3479–83. Epub 1978/07/01. 98769 10.1073/pnas.75.7.3479 PMC392801

[44] Jacob AE, Hobbs SJ. Conjugal transfer of plasmid-borne multiple antibiotic resistance in *Streptococcus faecalis* var. zymogenes. Journal of bacteriology. 1974;117(2):360–72. 4204433 10.1128/jb.117.2.360-372.1974 PMC285522

[45] Maguin E, Duwat P, Hege T, Ehrlich D, Gruss A. New thermosensitive plasmid for gram-positive bacteria. Journal of bacteriology. 1992;174(17):5633–8. 1324906 10.1128/jb.174.17.5633-5638.1992 PMC206509

[46] Nieto C, Espinosa M. Construction of the mobilizable plasmid pMV158GFP, a derivative of pMV158 that carries the gene encoding the green fluorescent protein. Plasmid. 2003;49(3):281–5. 12749839 10.1016/s0147-619x(03)00020-9

