# Supplementary figures and images for: Bacterial size matters: Multiple mechanisms controlling septum cleavage and diplococcus formation are critical for the virulence of the opportunistic pathogen Enterococcus faecalis
Source: PLoS Pathog. 2017 Jul 24;13(7):e1006526. doi: 10.1371/journal.ppat.1006526 (PMC5542707; doi:10.1371/journal.ppat.1006526)

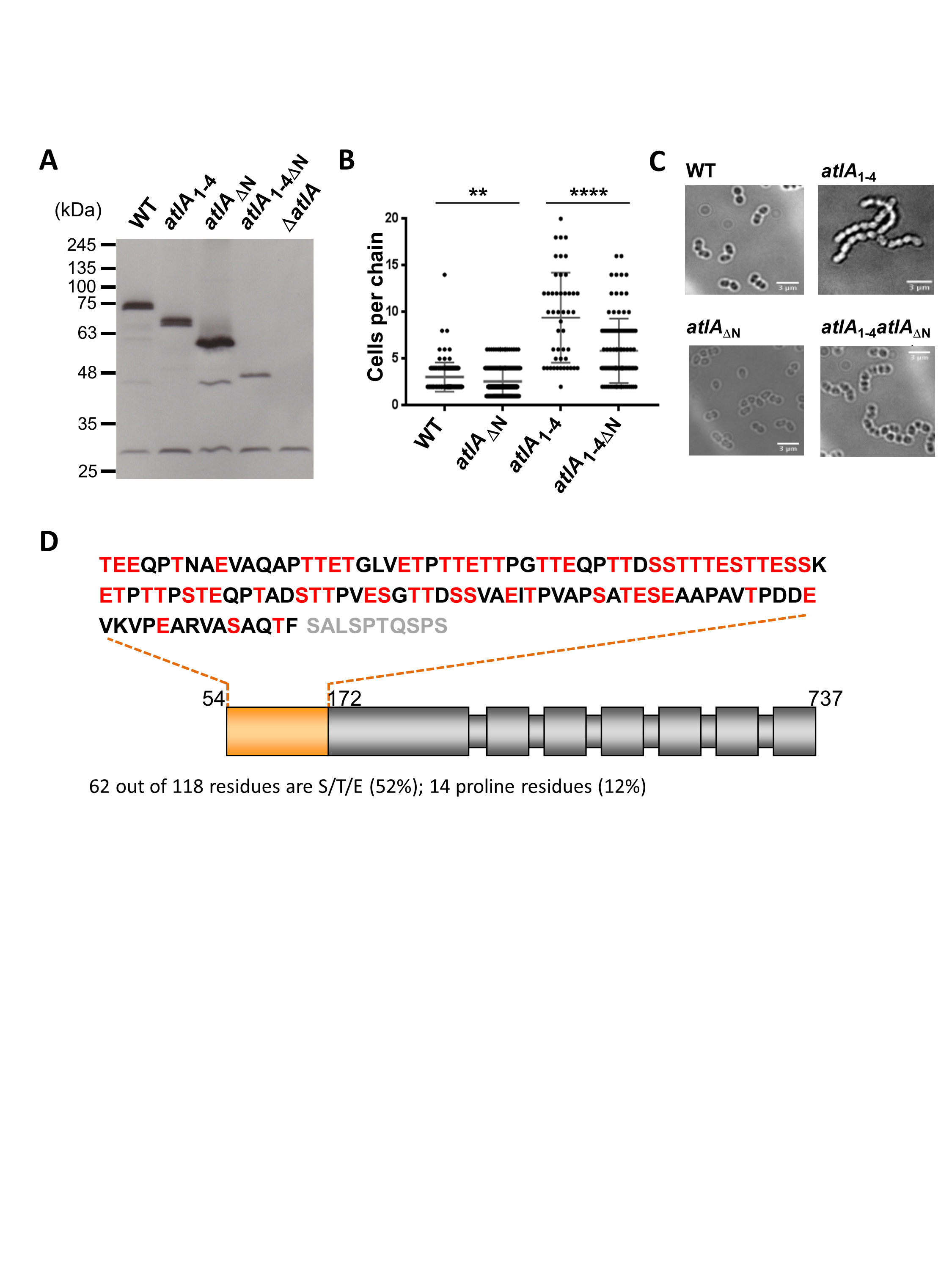

Supplement: S1 Fig — A. Western blot detection of AtlA proteins in culture supernatants. Supernatant proteins from exponentially growing cells were recovered by centrifugation, precipitated with 10% (m/v) TCA, washed with acetone and resuspended in PBS. Following SDS-PAGE and transfer on a nitrocellulose membrane, AtlA proteins were detected using an anti-AtlA polyclonal serum against the catalytic domain of AtlA. WT, E. faecalis JH2-2; atlAΔN, derivative expressing AtlA truncated from the N-terminal domain; atlA1-4, derivative expressing AtlA truncated from the two C-terminal LysM modules; AtlA1-4ΔN, AtlA1-4 truncated from the N-terminal domain WT; A strain with an in-frame deletion of atlA (ΔatlA) was used as a negative control. B. Average numbers of cells per chain formed by WT (3.0 ± 1.6; n = 427 cells); atlAΔN (2.6 ± 1.4; n = 534 cells); atlA1-4 (9.4 ± 4.8; n = 442 cells) and atlA1-4ΔN (5.9 ± 3.4; n = 610 cells) strains; ****P<0.0001. C. Light microscopy images showing cell chain lengths of the mutants. D. Sequence of AtlA N-terminal domain (residues 54 to 172). S/T/E residues are indicated in red. (TIF) [file ppat.1006526.s001.tif]

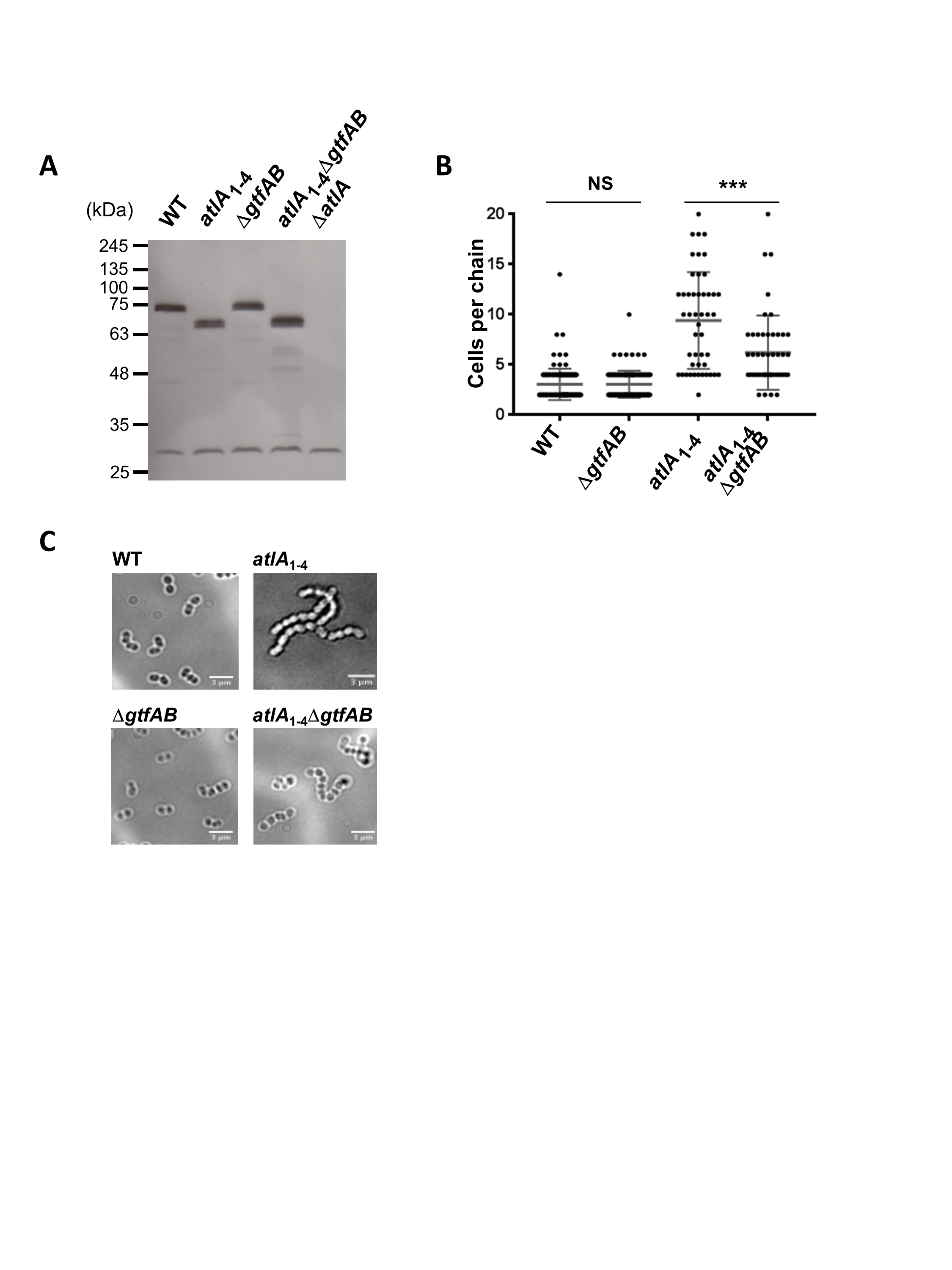

Supplement: S2 Fig — A. Western blot detection of AtlA proteins in culture supernatants (as described in S1 Fig). WT, E. faecalis JH2-2; atlA1-4, derivative expressing AtlA truncated from the two C-terminal LysM modules; ΔgtfAB, derivative with an in-frame deletion of the ΔgtfAB operon; atlA1-4ΔgtfAB, ΔgtfAB derivative with the truncation of the two C-terminal LysM modules of atlA. A strain with an in-frame deletion of atlA, ΔatlA was used as a negative control. B. Average numbers of cells per chain formed by WT (3.0 ± 1.6; n = 427 cells); ΔgtfAB (3.0 ± 1.3; n = 364 cells); atlA1-4 (9.4 ± 4.8; n = 442 cells); ΔgtfAB atlA1-4 (6.5 ± 3.7; n = 298 cells); NS, P>0.05; ***, P = 0.0005. C. Light microscopy images showing cell chain lengths of the mutants. (TIF) [file ppat.1006526.s002.tif]

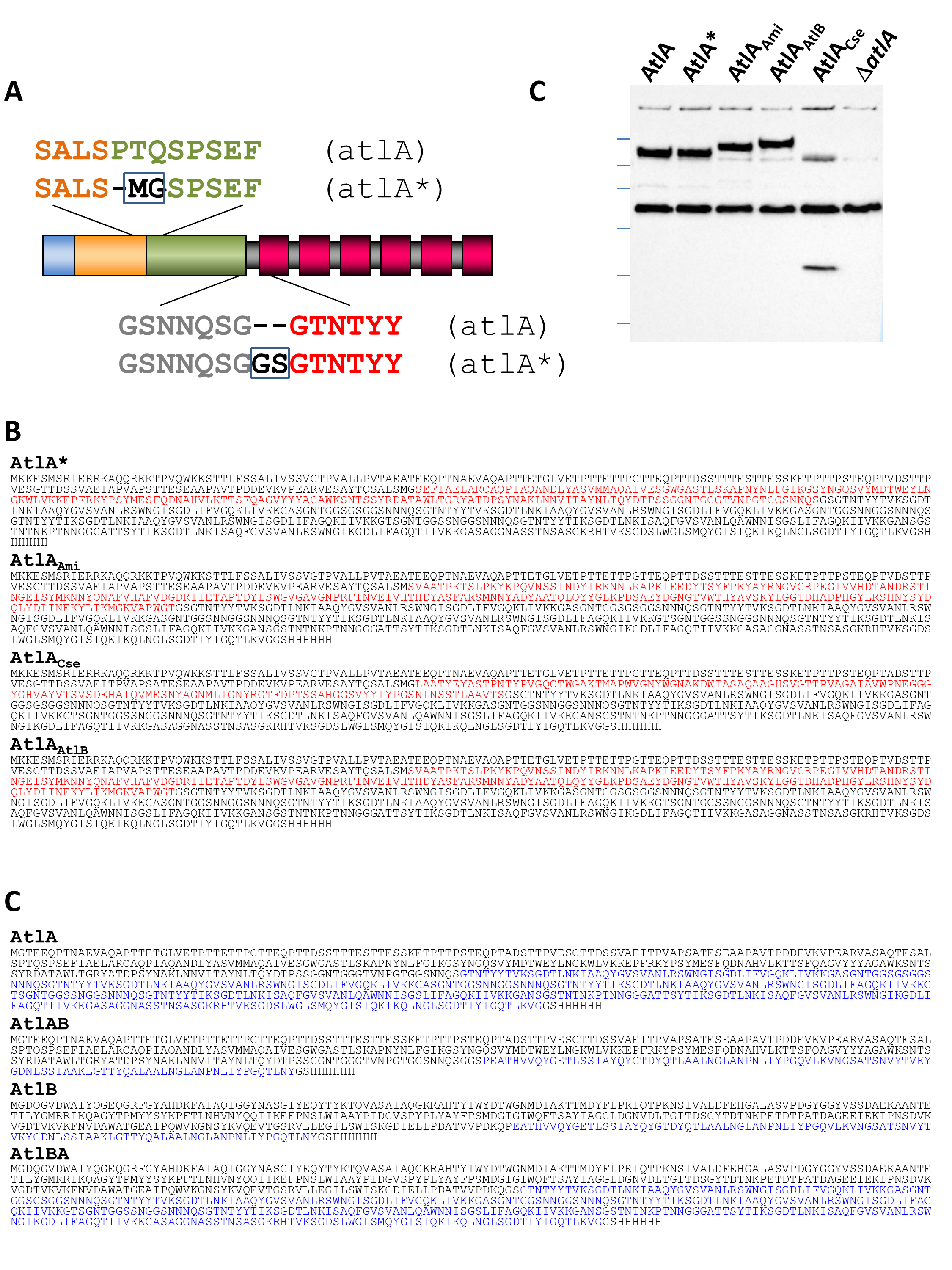

Supplement: S3 Fig — A. Schematic representation of the amino acid modifications introduced on either side of the catalytic domain for cloning purposes. B. Sequence of the AtlA variants with a swapped catalytic domain. Sequences in red correspond to catalytic domains of chimeric proteins expressed by recombinant E. faecalis strains analyzed in Fig 6. C. Western blot detection of chimeric proteins. Protein samples corresponding to crude extracts were run on an SDS-PAGE, transferred on a nitrocellulose membrane and probed with an anti-LysM polyclonal serum. The arrowheads indicate unspecific signals. D. Sequences in blue correspond to LysM domains used to construct the chimeric recombinant proteins expressed in E. coli (see Fig 5). (TIF) [file ppat.1006526.s003.tif]

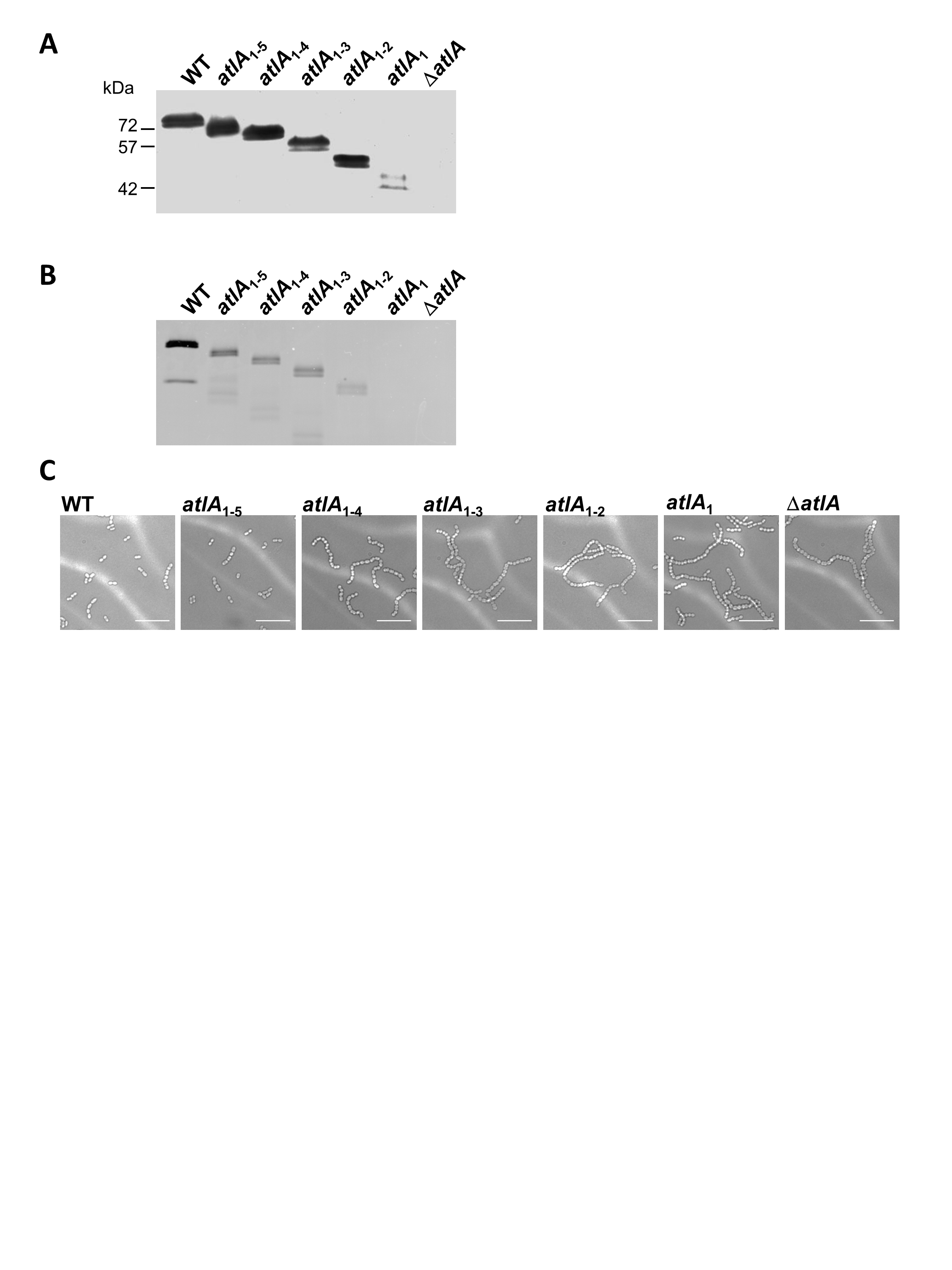

Supplement: S4 Fig — A. Western blot detection of AtlA proteins in culture supernatants. Cells were grown until exponential phase (OD600 = 0.2–0.5) and spun down. Supernatants were precipitated with 10% (m/v) TCA prior to detection of AtlA as described in supplementary Fig 1. Bands with the expected molecular weights were detected in all the strains. B. Zymogram analysis of AtlA activity in culture supernatants. Samples analyzed in (A) were loaded on an SDS-PAGE containing autoclaved M. luteus cells (OD600 = 2). After migration, the gel was rinsed and incubated in renaturing buffer to detect AtlA activity. Truncation of LysM repeats was associated with a decrease in AtlA activity. C. Light microscopy images showing cell chain lengths of the mutants. (TIF) [file ppat.1006526.s004.tif]

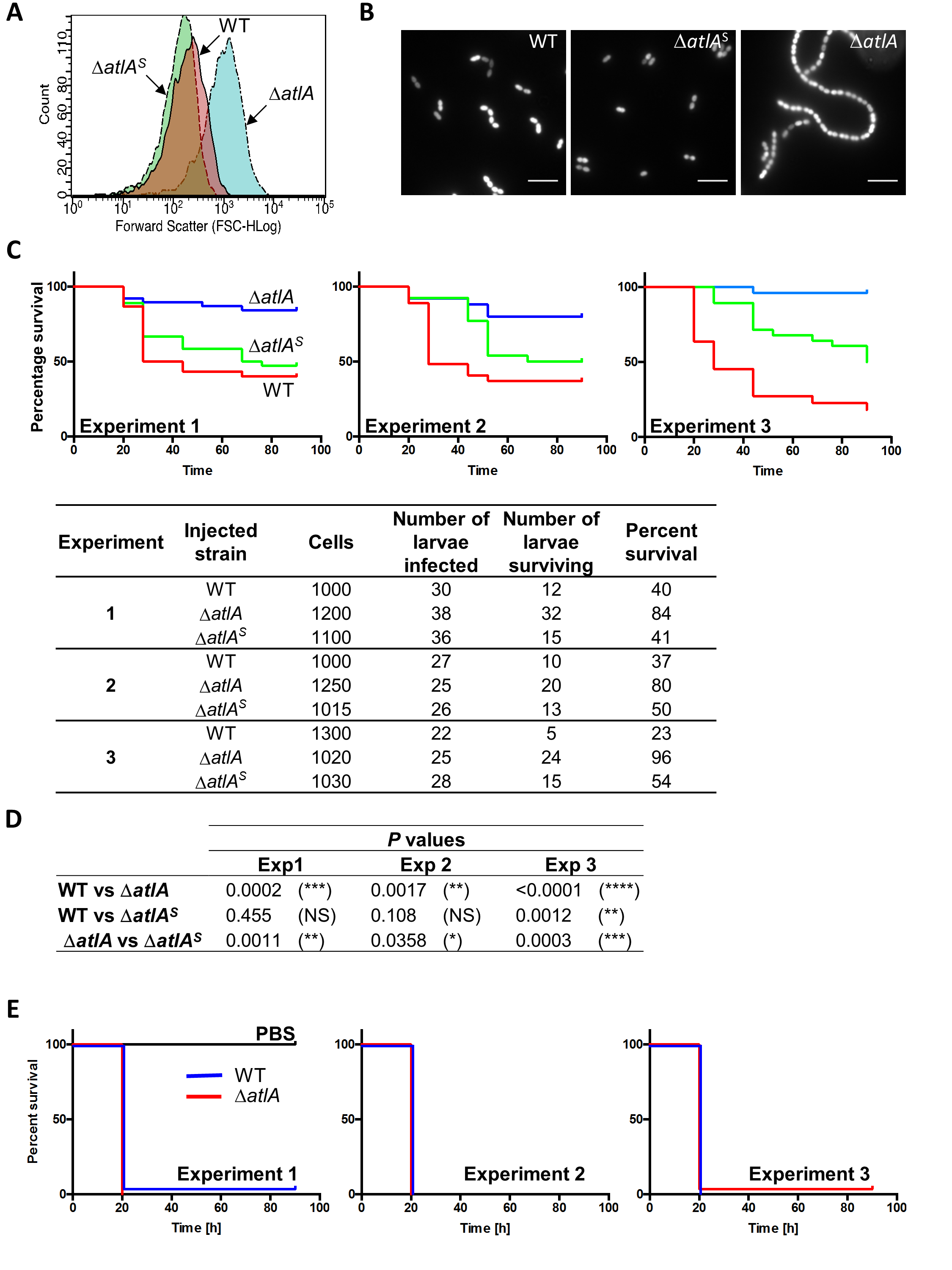

Supplement: S5 Fig — A. Comparison of median forward scattered (FSC) light values corresponding to the cell chain lengths of WT (OG1RF), ΔatlA and sonicated ΔatlA (ΔatlAS) strains. B. Light microscopy images showing cell chain lengths of WT and ΔatlA derivatives expressing cytoplasmic GFP. C. Survival of zebrafish larvae (n>20) following infection with E. faecalis OG1RF (WT) and atlA isogenic deletion mutant before (ΔatlA) and after (ΔatlAS) sonication to disperse long chains. The results corresponding to three independent experiments are shown. For each experiment, the number of cells injected (determined after sonication) is indicated. D. P values resulting from pairwise comparisons using the log rank test. E. survival of phagocyte-depleted zebrafish larvae following injection with 1250 cells of E. faecalis OG1RF (WT) or ΔatlA. (TIF) [file ppat.1006526.s005.tif]

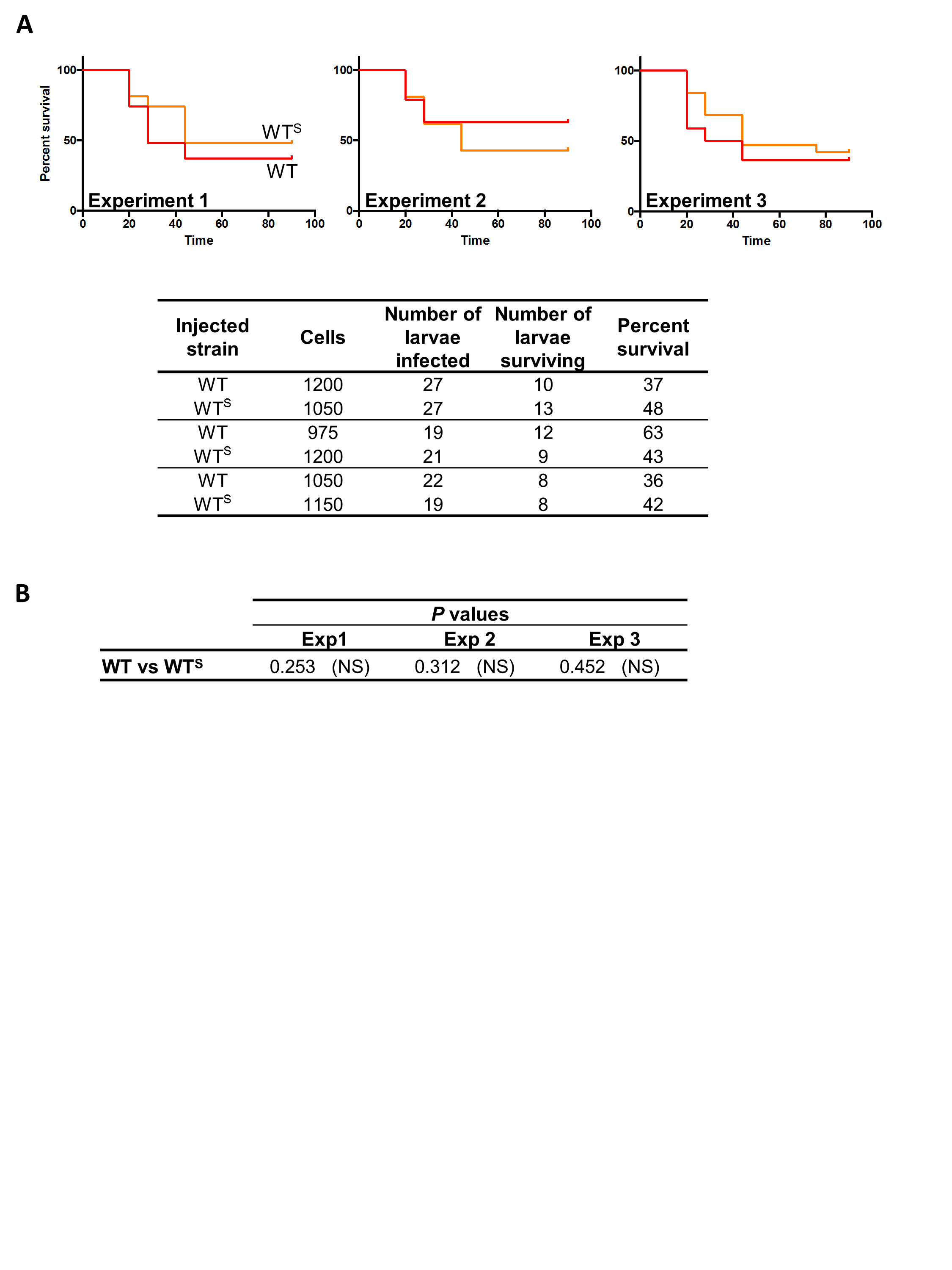

Supplement: S6 Fig — A. Survival of zebrafish larvae (n>20) following infection with E. faecalis OG1RF (WT) and OG1RF sonicated (WTS) cells. The results corresponding to three independent experiments are shown. For each experiment, the number of cells injected (determined after sonication) is indicated. B. P values resulting from pairwise comparisons using the log rank test. (TIF) [file ppat.1006526.s006.tif]

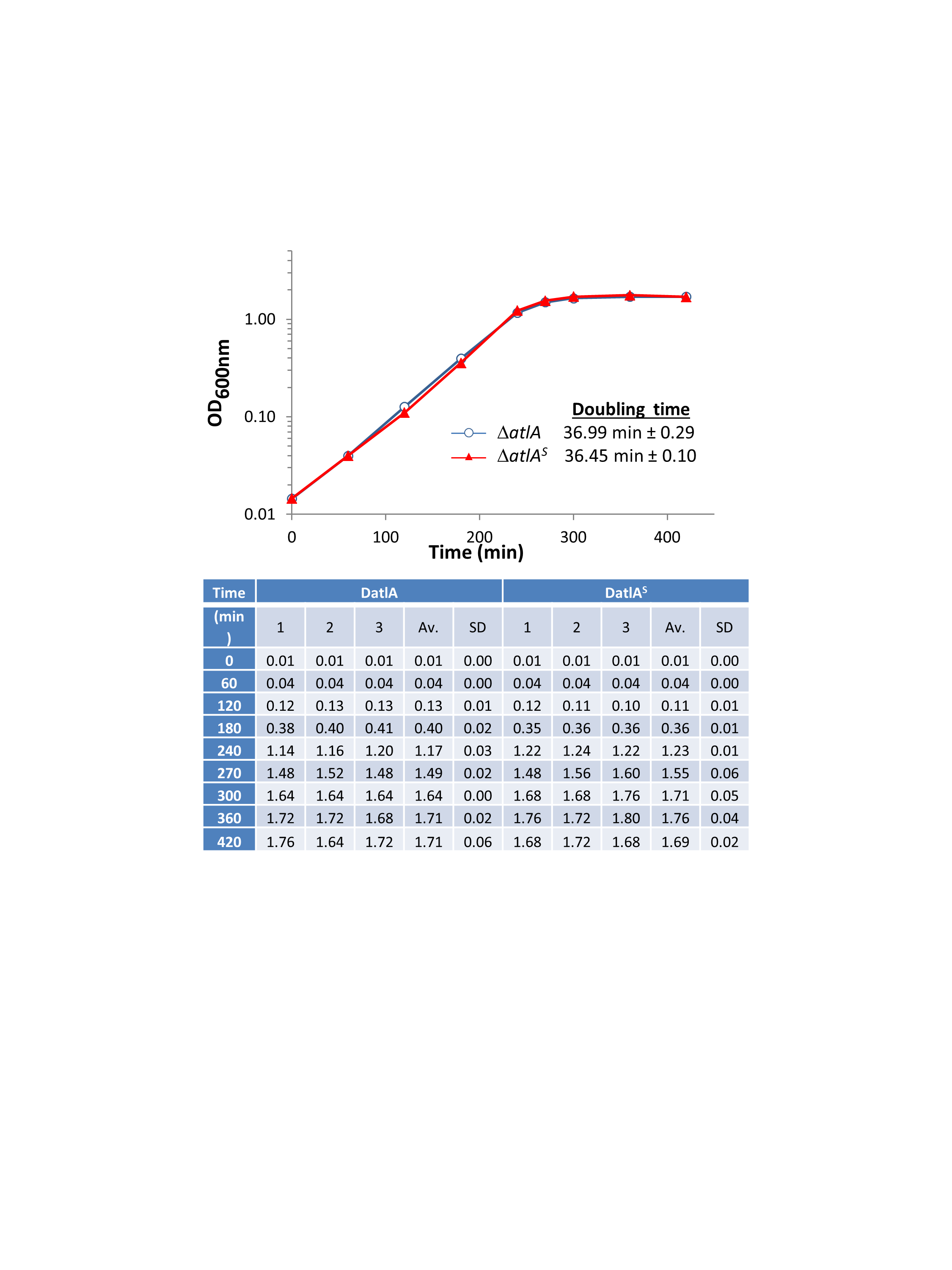

Supplement: S7 Fig — Cells from an overnight culture in BHI were diluted to an OD600 of 0.01 in 25ml BHI and growth of standing cultures were monitored over 7 hours. The growth rate of each strain was determined using the OD values between 60 and 240 minutes (exponential growth). The data presented are the average of 3 independent cultures. OD values of individual growth curves are presented. (TIF) [file ppat.1006526.s007.tif]
