# Supplementary material for: Bacterial size matters: Multiple mechanisms controlling septum cleavage and diplococcus formation are critical for the virulence of the opportunistic pathogen Enterococcus faecalis
Source: PLoS Pathog. 2017 Jul 24;13(7):e1006526. doi: 10.1371/journal.ppat.1006526 (PMC5542707; doi:10.1371/journal.ppat.1006526)
Supplement: S1 Table — (DOC) [file ppat.1006526.s008.doc]

SUPPLEMENTARY TABLE 1. Oligonucleotides used in this study

**Oligonucleotides** ^a^ (Sequence 5’🠦3’) restriction site plasmid

AtlATEV_H11 aaaccatgggaACAGAAGAGCAGCCAACAAATGC NcoI pET-AtlA_TEV_

AtlATEV_H12 TGATTGAAAATATAAATTTTCTGTTACTCTAGCTTCTGGTACTTTTACTT pET-AtlA_TEV_

AtlATEV_H21 GTAACAGAAAATTTATATTTTCAATCAGCGTTATCACCGACGCAAAGTCC pET-AtlA_TEV_

AtlATEV_H22 ttggatccACCAACTTTTAAAGTTTGACCAA BamHI pET-AtlA_TEV_

AtlB_1 aaaccATGGGTGACCAAGGCGTTGACTGGGCG NcoI pET-AtlB

AtlB_2 tttGGATCCATAATTCAATGTTTGGCCAGGATA BamHI pET-AtlB

AtlBA_H11 aaaccATGGGTGACCAAGGCGTTGACTGGGCG NcoI pET-AtlBA

AtlBA_H12 TTGCTTATTGGGAACAGTTGTAGCATCTGGCAACAATTCAATATCGCCT pET-AtlBA

AtlBA_H21 TGCTACAACTGTTCCCAATAAGCAAggaTCAGGAACGAACACGTACTATA pET-AtlBA

AtlBA_H22 tttGGATCCATAATTCAATGTTTGGCCAGGATA BamHI pET-AtlBA

AtlAB_H11 aaaccatgggaACAGAAGAGCAGCCAACAAATGC NcoI pET-AtlAB

AtlAB_H12 TAATGTCATTACCGCATATAACTTAACTCAATATGATACACCA pET-AtlAB

AtlAB_H21 CATATAACTTAACTCAATATGATACACCATCTTCTGGTGGAAATACTGGGG pET-AtlAB

AtlAB_H22 tttGGATCCATAATTCAATGTTTGGCCAGGATA BamHI pET-AtlAB

DeltaN_H11 tttCTCGAGTGATCTTTTGGATTTAATGCTT XhoI pGDN

DeltaN_H12 TGGCTGCTCTTCTGTTGCCTCAGCAGTCACTGGTAGTAACGC pGDN

DeltaN_H21 CTGCTGAGGCAACAGAAGAGCAGCCATCAGCGTTATCACCGACGCAAAG pGDN

DeltaN_H22 tttCTGCAGTCATATTGAGTTAAGTTATATGCGG PstI pGDN

AtlA*_H11 tttCTCGAGgaACAGAAGAGCAGCCAACAAATGC XhoI pGatlA*

AtlA*_H12 TAAATTCTGAAGGACTtcccatgGATAACGCTGAAAAAGTTTGCGCAGA NcoI pGatlA*

AtlA*_H21 CAGCGTTATCcatgggaAGTCCTTCAGAATTTATTGCCGAGTTAGCTCG NcoI pGatlA*

AtlA*_H22 TACGTggatccTCCTGATTGATTGTTCGAGCCGCCTGTTCCTGGATTAA BamHI pGatlA*

AtlA*_H31 CGGCTCGAACAATCAATCAGGAggatccACGTACTATACTGTAAAATC BamHI pGatlA*

AtlA*_H32 TTTGAATTCCGTAGGCTTGTTCGTATTCGTTGA EcoRI pGatlA*

AtlA-Cse_Fw tATCcatggTAGCAGCTACATACGAATATGCAT NcoI pGatlA-Cse

AtlA-Cse_Rev CGTggatccTGGATAAATATAATATACAGAACCACCATGCGCTG BamHI pGatlA-Cse

AtlA-AtlB_Fw ATCcatggGGAGACCAAGGTGTGGATTGGGCG NcoI pGatlA-AtlB

AtlA-AtlB_Rev CGTggatccTTGCTTATCAGGGACGACTGTTGC BamHI pGatlA-AtlB

AtlA-Ami_Fw TATCcatggtcTCAGTTGCTGCAACACCAAAAAC NcoI pGatlA-Ami

AtlA-Ami_Rev CGTggatccCGTACCCCAaGGCGCCACTTTACCCATTT BamHI pGatlA-Ami

BBH_H11 AAACTCGAGCAACTGGCGAAGCTATTCCAGATTG XhoI pGBBhis

BBH_H12 CTTATTTCTTAGTGGTGATGGTGATGATGATAATTCAATGTTTGGCCAGGA pGBBhis

BBH_H21 GAATTATCATCATCACCATCACCACTAAGAAATAAGTAAAAGACCTACTTCTC pGBBhis

BBH_H22 AAAGAATTCAGAAGCAATGCCTCTGCCTCTATAG EcoRI pGBBhis

ABH_Fw CCCCtcgagTTCGTTCCGTCAATTCAA XhoI pGABhis

ABH_Rev CAGGAATTCCTTGTCCAGATGGATAGGGAAACATAT EcoRI pGABhis

BBH_H11 CCCCtcgagCATATAACTTAACTCAATATGATACACC XhoI pGAAhis

BBH_H12 TAATGATGATGATGATGATGGGATCCaccaacttttaaagtttgaccaaTATA pGAAhis

BBH_H21 TCCCATCATCATCATCATCATTAAtttaagattaaaaaaaatagctatctttg pGAAhis

BBH_H22 ACCGAATTCccacatcataaccgccacgttcact EcoRI pGAAhis

1LysM_H11 GACCTCGAGTTTTTCAGCGTTATCACCGACGC XhoI pGatlA1

1LysM_H12 TAATGATGATGATGATGATGGGATCCaccaacttttaaagtttgaccaaTATA pGatlA1

1LysM_H21 TTTAAGATTGAAAAAAATAGCTATCTTTGGTAACATGAG pGatlA1

1LysM_H22 TTGGGTACCACCTAAATTCGTCAAAGAAACGGTC KpnI pGatlA1

2LysM_H11 GACCTCGAGTTTTTCAGCGTTATCACCGACGC XhoI pGatlA1-2

2LysM_H12 CTTAAATTATCAAGATCCTTTTTTCACGATGAGTTTTTGACC pGatlA1-2

2LysM_H21 AAGGATCTTGATAATTTAAGATTGAAAAAAATAGCTATC pGatlA1-2

2LysM_H22 TTGGGTACCACCTAAATTCGTCAAAGAAACGGTC KpnI pGatlA1-2

3LysM_H11 GACCTCGAGTTTTTCAGCGTTATCACCGACGC XhoI pGatlA1-3

3LysM_H12 CAATCTTAAATTATCAAGATCTTTTTTTCACAATAATTTTTTGACCAGCGAAG pGatlA1-3

3LysM_H21 GATCTTGATAATTTAAGATTGAAAAAAATAGCTATCTTTGGTAACATGAG pGatlA1-3

3LysM_H22 TTGGGTACCACCTAAATTCGTCAAAGAAACGGTC KpnI pGatlA1-3

4LysM_H11 GACCTCGAGTTTTTCAGCGTTATCACCGACGC XhoI pGatlA1-4

4LysM_H12 CTTAAATTATCAAGATCTTTTTTTCACGATAATCTTTTGACCAGCAAAAATC pGatlA1-4

4LysM_H21 TCGTGAAAAAAAGATCTTGATAATTTAAGATTGAAAAAAATAGCTATCTTTG pGatlA1-4

4LysM_H22 TTGGGTACCACCTAAATTCGTCAAAGAAACGGTC KpnI pGatlA1-4

5LysM_H11 GACCTCGAGTTTTTCAGCGTTATCACCGACGC XhoI pGatlA1-5

5LysM_H12 TTCAATCTTAAATTATCAAGATCTTTTTTTCACGATGATTGTTTGACCAGC pGatlA1-5

5LysM_H21 GATCTTGATAATTTAAGATTGAAAAAAATAGCTATCTTTGGTAACATGAG pGatlA1-5

5LysM_H22 TTGGGTACCACCTAAATTCGTCAAAGAAACGGTC KpnI pGatlA1-5
